# The link between GABA levels and P300 abnormalities in schizophrenia spectrum disorders: regional and symptom-based insights

**DOI:** 10.1038/s41537-026-00730-5

**Published:** 2026-01-23

**Authors:** Berkhan Karslı, Verena Meisinger, Genc Hasanaj, Marcel S. Kallweit, Fanny Dengl, Gizem Vural, Julian Melcher, Maxim Korman, Nicole Klimas, Susanne Schmölz, Antonia Šušnjar, Alexandra Hisch, Lenka Krčmář, CDP Working Group, Berkhan Karslı, Verena Meisinger, Genc Hasanaj, Marcel S. Kallweit, Fanny Dengl, Julian Melcher, Maxim Korman, Nicole Klimas, Susanne Schmölz, Alexandra Hisch, Lenka Krčmář, Emanuel Boudriot, Joanna Moussiopoulou, Vladislav Yakimov, Oliver Pogarell, Andrea Schmitt, Peter Falkai, Thomas Geyer, Lukas Roell, Elias Wagner, Florian J. Raabe, Daniel Keeser, Emanuel Boudriot, Joanna Moussiopoulou, Vladislav Yakimov, Oliver Pogarell, Andrea Schmitt, Peter Falkai, Thomas Geyer, Lukas Roell, Elias Wagner, Florian J. Raabe, Daniel Keeser

**Affiliations:** 1 Department of Psychiatry and Psychotherapy, LMU University Hospital, LMU Munich, Munich, Germany; 2 NeuroImaging Core Unit Munich (NICUM), LMU University Hospital, LMU Munich, Munich, Germany; 3 Evidence-based Psychiatry and Psychotherapy, Faculty of Medicine, University of Augsburg, Augsburg, Germany; 4 Department of Psychology, Ludwig Maximilian University, Munich, Germany; 5 Athinoula A. Martinos Center for Biomedical Imaging, Institute for Innovation in Imaging, Department of Radiology Massachusetts General Hospital and Harvard Medical School, Boston, MA USA; 6 Max Planck Institute of Psychiatry, Munich, Germany; 7 International Max Planck Research School for Translational Psychiatry (IMPRS-TP), Munich, Germany; 8 German Center for Mental Health (DZPG), Partner Site Munich/Augsburg, Munich, Germany; 9 Laboratory of Neuroscience (LIM27), Institute of Psychiatry, University of São Paulo, São Paulo, Brazil; 10 Department of Psychiatry, Psychotherapy and Psychosomatics, Medical Faculty, University of Augsburg, BKH Augsburg, Augsburg, Germany; 11 Munich Center for Neurosciences (MCN), LMU Munich, Planegg-Martinsried, Germany

**Keywords:** Schizophrenia, Neural circuits

## Abstract

According to the excitation-inhibition imbalance theory, GABAergic and glutamatergic systems influence the clinical symptoms, particularly cognitive deficits in schizophrenia spectrum disorders (SSD). These systems have been found disrupted in the anterior cingulate cortex (ACC) and dorsolateral prefrontal cortex (DLPFC) in SSD, and may contribute to P300 abnormalities in electroencephalography recordings. Therefore, we explored the relationships among MRS-derived GABA and Glx levels in the ACC and left DLPFC (lDLPFC), auditory P3b subcomponent amplitudes and latencies, cognition, and symptom severity in SSD. In total, 107 patients and 107 healthy controls (HC) were included in the study, with the exact numbers varying across specific analyses. We grouped patients into higher (SSD+, *N* = 41) and lower (SSD−, *N* = 65) symptom severity clusters based on PANSS total scores. P3b amplitudes were lower in SSD patients than HC at central and parietal sites. SSD+ exhibited widespread P3b amplitude reductions, significant at parietal and trend-level at central and frontal regions, while SSD− showed a trend-level amplitude reduction limited to the parietal region. GABA levels in the lDLPFC were higher in SSD− compared to controls and were positively associated with P3b amplitudes at central and parietal sites within SSD− and overall SSD group. Although P3b amplitudes positively correlated with the BACS composite scores and behavioral performance, lDLPFC GABA levels showed no direct association with cognitive or behavioral performance. ACC GABA, ACC Glx, and lDLPFC Glx levels showed no group differences or P3b associations. Our findings suggest P3b amplitude reductions as a marker of cognitive dysfunction in SSD, more pronounced in patients with higher illness severity, and that enhanced lDLPFC GABA may contribute to offsetting these reductions. Our work provides the first empirical evidence of the interplay between the GABAergic system and cortical electrophysiological signal patterns associated with cognitive dysfunction in SSD.

## Introduction

Schizophrenia spectrum disorders (SSD) are complex neuropsychiatric disorders characterized by positive symptoms such as hallucinations and delusions, negative symptoms such as anhedonia and avolition, and cognitive impairment^1^. Cognitive deficits, which are directly correlated with patients’ daily functioning, remain largely unresponsive to current treatments that primarily target dopamine D2 receptors^2^, perhaps due to a limited understanding of the underlying mechanisms. One of the most consistently replicated neural correlates of SSD-related cognitive impairments is P300 amplitude reduction^3,4^. P300 is an event-related potential (ERP) component, typically elicited as a positive peak around 300 ms after presenting an infrequent target stimulus within a series of standard stimuli in an oddball task, was shown to reflect a cognitive process^5^. Importantly, the P300 consists of two subcomponents, each of them representing different cognitive processes and neural generators. The P3a is elicited by infrequent, novel, or salient stimuli that do not require a response. It is typically maximal at fronto-central sites, peaking around 250–300 ms after stimulus presentation, and reflects bottom-up attentional orienting to novelty^5–8^. In contrast, the P3b is elicited by infrequent target stimuli that require a voluntary response (e.g., a button press or mental counting) and was the focus of the current study. It reflects top-down attention and memory updating and is maximal at parietal sites, peaking around 300–350 ms after stimulus presentation^5,8,9^. Both P3a and P3b amplitude reductions in SSD are more pronounced with higher clinical symptom severity and fluctuate with longitudinal changes in symptom levels, becoming greater during symptom deterioration and milder when symptoms improve^10,11^.

Distinct neural generators have been proposed for the two P300 subcomponents. The P3a is primarily associated with activation in the hippocampus, dorsolateral prefrontal cortex (DLPFC), cingulate gyrus, and supramarginal gyrus, whereas the P3b has been linked to the temporoparietal junction, ventral temporo-frontal regions, and hippocampus^9,12,13^. However, accumulating evidence suggests that these regions form an interconnected network. In particular, the anterior cingulate cortex (ACC), DLPFC, and temporoparietal cortices jointly contribute to a distributed P300-generating system^14,15^. The DLPFC and ACC are involved in higher-order cognitive functions, including working memory, cognitive control, attention allocation, and conflict monitoring^16–18^. In SSD, structural and functional abnormalities in these regions (e.g., gray and white matter reductions, hypofunction) have been consistently reported and are linked to impairments in these cognitive domains^19–21^. These impairments likely contribute to the reduced P300 amplitude often observed in SSD. For instance, ACC hypoactivation during P3b tasks in SSD is associated with diminished regulation of stimulus relevance^22^, and experimental inhibition of bilateral DLPFC reduces P300-related amplitude to task-relevant stimuli^23^. Together, these findings suggest that DLPFC and ACC dysfunctions may underlie the P300 amplitude reductions seen in SSD by impairing cognitive control, monitoring, and adaptive behavioral responses.

Neurochemical imbalances, especially disruptions in the excitation-inhibition (E/I) balance mediated by glutamate and gamma-aminobutyric acid (GABA), have been suggested to play a crucial role in the pathophysiology of SSD^24^. Such disruptions can lead to neural circuit dysfunctions contributing to cognitive deficits^5^. Recent meta-analyses using proton magnetic resonance spectroscopy (¹H-MRS), positron emission tomography (PET), and single-photon emission computed tomography (SPECT) have identified altered glutamatergic and GABAergic metabolite concentrations across brain regions. The medial prefrontal cortex (mPFC) shows consistent reductions in glutamate in SSD^25–28^, and decreased GABA has been observed in the midcingulate cortex (MCC) but not in the ACC^25,29^. The DLPFC is another relevant region, given its role in cognitive control and evidence from animal models showing disrupted glutamatergic and GABAergic signaling^30,31^. However, human neuroimaging findings in the DLPFC have been mixed. Some meta-analyses found no significant group differences^27,32^, while others report elevated glutamate and lower variability in antipsychotic-naïve patients, with medicated patients showing increased variability but no elevation^33^. Moreover, pharmacological studies in healthy individuals show that disrupting glutamatergic and GABAergic systems reduces P300 amplitude, paralleling patterns seen in SSD (see Hamilton et al.^5^ for a review).

The present study aims to investigate the role of the GABAergic and glutamatergic systems in P3b abnormalities and related cognitive deficits in SSD, while accounting for symptom heterogeneity. Although several neurotransmitter systems have been implicated in P300 modulation in SSD^5^, prior MRS studies have reported associations primarily in healthy samples. For example, Hall et al.^34^ found that ACC Gln/Glu ratio and Gln, but not Glu or Glx, were associated with frontal P3a, while no glutamatergic measures from the parietal–occipital cortices were linked to parietal P3b. Similarly, Gallinat et al.^35^ reported an association between hippocampal glutamate levels and frontal P3b theta activity during stimulus processing. To our knowledge, however, no study has directly examined whether GABA or Glx levels in frontal cortical regions are associated with P3b amplitude reductions in SSD. First, we sought to replicate the well-established P3b amplitude reductions in patients. Second, to account for symptom heterogeneity and its previously reported association with P3b amplitude reductions^10,11^, we clustered patients based on symptom severity, hypothesizing that more symptomatic patients would show greater reductions. Third, we examined whether GABA and Glx (a composite of glutamate and glutamine) levels in the ACC and DLPFC differ between healthy controls, the overall patient group, and patient clusters. Fourth, we examined whether these metabolite levels are associated with P3b amplitude reductions in the overall patient group and in patient clusters. Finally, we examined how metabolite levels and disrupted P3b indices are each related to cognition and behavioral task performance in the patient group and clusters. These findings may help clarify the neurochemical contributions to P3b abnormalities and their impact on cognition in SSD.

## Methods

### Study sample and design

All 214 participants (107 patients with Schizophrenia Spectrum Disorder [SSD] and 107 healthy controls [HC]) included in the study were recruited as part of the Clinical Deep Phenotyping (CDP) study^36^, a project within the Munich Mental Health Biobank^37^ (see Table 1). While the broader P300-generating network involves multiple cortical and subcortical regions, analyses in the present study were limited to the anterior cingulate cortex (ACC) and left dorsolateral prefrontal cortex (lDLPFC), in accordance with the spectroscopy data available within the predefined scope of the parent project. As available data varied across modalities, group-level descriptives for each measure are provided in Table 2, and detailed sample size breakdowns for each analysis are reported in Supplementary Table 5. The study, approved by the Ethics Committee of the Faculty of Medicine, LMU Munich (project numbers: 20-0528 and 22-0035) and registered in the German Clinical Trials Register (DRKS; registration ID: DRKS00024177), included individuals aged 18–65 years recruited from the in- and outpatient population of the Department of Psychiatry and Psychotherapy at the LMU University Hospital, along with healthy controls. Informed consent was obtained from all participants. The majority of patients were treated with second-generation antipsychotic medications. Inclusion criteria for patients were a diagnosis of schizophrenia (SZ), schizoaffective disorder (SZA), brief psychotic disorder (BrPsyD), or delusional disorder (DD) according to the MINI International Neuropsychiatric Interview^38^. General exclusion criteria included any primary psychiatric disorder other than those listed, clinically relevant central nervous system (CNS) disorders (past or present), pregnancy, major language barriers, and substance abuse (except alcohol, nicotine, and caffeine) more than once during the last 12 months. Symptom severity was assessed by trained staff using the Positive and Negative Syndrome Scale (PANSS)^39^. Cognitive function was assessed using the Brief Assessment of Cognition in Schizophrenia (BACS)^40^, and z-standardized BACS composite scores were calculated. Detailed information about the assessments and score calculations is included in the Supplemental Methods. All participants completed one P3b, one MRS, and one set of clinical and cognitive assessments in separate sessions, with no repeated or longitudinal measurements. The interval between P3b and MRS sessions was on average 3.56 days (median = 0, range = 0–75 days). To address potential concerns regarding session timing, we replicated the main P3b-MRS results in the subgroup of participants who completed both measurements on the same day, and the findings remained unchanged (see Supplemental Results).

**Table 1:** Sample characteristics table.

	SSD		HC		*p*
	Mean ± SD or *n* (%)	*n*	Mean ± SD or n (%)	*n*	
*Demographic characteristics*					
Age, Years	38.66 ± 12.25	107	33.93 ± 12.22	107	0.005^a^
Sex, Female:Male (% Female)	32:75 (29.9%)		55:52 (51.4%)		0.002^b^
BMI	28.44 ± 5.66	105	23.42 ± 3.35	105	<0.001^a^
Smoking, Yes:No (% Yes)	53:52 (50.5%)		8:95 (7.8%)		<0.001^b^
*Comorbidities*					
Diabetes, Yes:No (% Yes)	4:103 (3.7%)		1:106 (0.9%)		0.369^b^
Hypertension, Yes:No (% Yes)	17:90 (15.9%)		4:103 (3.7%)		0.005^b^
*Illness characteristics*					
Illness Duration, Months	142.63 ± 121.92	105			
Antipsychotic Treatment Duration, Months	123.62 ± 112.76	95			
Benzodiazepine Use, Yes:No (% Yes)	13:94 (12.1%)				
CPZeq, mg	343.22 ± 249.21	104			
Remission Andreasen, Yes:No (% Yes)	51:55 (48.1%)				
PANSS Positive Symptoms	12.64 ± 5.28	106	7.26 ± 0.78	107	<0.001^a^
PANSS Negative Symptoms	13.42 ± 5.71	106	7.42 ± 0.97	107	<0.001^a^
PANSS General Symptoms	28.97 ± 8.9	106	17.06 ± 1.55	107	<0.001^a^
PANSS Total Score	55.03 ± 17.3	106	31.74 ± 2.64	107	<0.001^a^
GAF	53.42 ± 11.37	104	89.9 ± 6.49	107	<0.001^a^
BACS Composite z Score	−1.84 ± 1.63	99	0 ± 1	104	<0.001^a^
**Diagnosis**					
Schizophrenia	67 (62.6%)				
Schizoaffective Disorder	30 (28.0%)				
Brief Psychotic Disorder	5 (4.7%)				
Unspecified SSD	4 (3.7%)				
Delusional Disorder	1 (0.9%)				

*HC* healthy control participant, *SSD* schizophrenia spectrum disorder, *BACS* Brief Assessment of Cognition in Schizophrenia, *BMI* body mass index, *CPZeq* chlorpromazine equivalent dose, *GAF* Global Assessment of Functioning, *PANSS* Positive and Negative Syndrome Scale.

^a^Welch’s *t* test.

^b^Fisher’s exact test.

**Table 2:** Breakdown of group-level descriptives (mean ± SD) and sample sizes (N) across HC (healthy controls), SSD (schizophrenia spectrum disorder), SSD− (lower-symptom cluster), and SSD+ (higher-symptom cluster) for magnetic resonance spectroscopy (MRS), P3b measures, and cognitive/behavioral measures.

	HC	SSD	SSD −	SSD +
Measure and region	*N* (Mean ± SD)	*N* (Mean ± SD)	*N* (Mean ± SD)	*N* (Mean ± SD)
*MRS*				
ACC GABA (mM)	33 (1.39 ± 0.33)	29 (1.48 ± 0.54)	14 (1.48 ± 0.49)	14 (1.48 ± 0.61)
lDLPFC GABA (mM)	51 (1.25 ± 0.37)	47 (1.39 ± 0.39)	37 (1.43 ± 0.39)	10 (1.26 ± 0.41)
ACC Glx (mM)	51 (9.19 ± 3.43)	49 (8.75 ± 2.95)	23 (8.59 ± 1.88)	25 (9.01 ± 3.71)
lDLPFC Glx (mM)	51 (8.52 ± 1.37)	48 (8.41 ± 1.97)	38 (8.1 ± 1.92)	10 (9.61 ± 1.73)
*P3b*				
Frontal amplitude (uV)	101 (6.47 ± 4.12)	102 (5.49 ± 3.77)	62 (6.22 ± 4.09)	39 (4.31 ± 2.93)
Central amplitude (uV)	104 (9.76 ± 5.85)	106 (7.65 ± 4.57)	65 (8.41 ± 4.6)	40 (6.48 ± 4.35)
Parietal amplitude (uV)	106 (11.4 ± 6.09)	107 (7.93 ± 4.01)	65 (8.52 ± 3.89)	41 (7.05 ± 4.12)
Frontal latency (ms)	101 (344.87 ± 54.23)	102 (338.5 ± 53.35)	62 (337.95 ± 52.87)	39 (338.34 ± 55.09)
Central latency (ms)	104 (353.59 ± 50.02)	106 (344.01 ± 51.57)	65 (343.99 ± 47.7)	40 (343.26 ± 58.32)
Parietal latency (ms)	106 (346.44 ± 37.45)	107 (353.9 ± 42.54)	65 (354.27 ± 37.35)	41 (353.66 ± 50.57)
*Cognition and Behavior*				
BACS composite score (z-score)	104 (0.02 ± 1)	99 (-1.84 ± 1.63)	61 (-1.55 ± 1.55)	38 (-2.32 ± 1.65)
Task accuracy (%)	107 (98.65 ± 2.95)	107 (95.67 ± 10.83)	65 (95.74 ± 10.01)	41 (95.56 ± 12.28)
Reaction time (ms)	107 (338.81 ± 78.61)	107 (386.17 ± 92.85)	65 (386.28 ± 94.88)	41 (384.69 ± 91.46)

*ACC* anterior cingulate cortex, *BACS* Brief Assessment of Cognition in Schizophrenia, *GABA* gamma-aminobutyric acid, *Glx* glutamate + glutamine, *lDLPFC* left dorsolateral prefrontal cortex, *mM* millimolar, *ms* millisecond, *µV* microvolt.

### P3b recording and preprocessing

The P3b was recorded using an Electro-Cap (Electro-Cap International, Inc, Eaton, OH, USA) following the International 10–20 system, connected to a 32-channel BrainAmp amplifier (Brain Products, Martinsried, Germany) in a quiet, dimly lit cabin at the EEG department at the Department of Psychiatry and Psychotherapy, University Hospital LMU Munich. Participants performed an auditory oddball task wearing Phillips headphones, pressing a button in response to infrequent target tones (2000 Hz, 85 dB, 20% of 700 trials) randomly placed among standard tones (1000 Hz, 80 dB, 80% of trials) while keeping their eyes closed for 18 min. All tones (tone duration 40 ms with 10-ms rise/fall times, 1.5-s interstimulus interval) were generated via Presentation software (neurobs.com; v14.9). Prior to the task, participants were asked about potential hearing problems and tested for their ability to discriminate the tones used in the task; only those able to do so were included.

EEG data were processed using MATLAB (The Mathworks Inc.) with EEGLAB (v2022.0)^41^, following a pipeline adapted from Adams et al.^42^, which offers a systematic multi-step artifact rejection approach and stringent data quality control criteria. The pipeline was modified for the present study to ensure its suitability for P3b analysis. Specifically, we adjusted the band-pass filter (0.05–70 Hz), extended the epoching window (−500 to 1000 ms), and added a final ±100 µV amplitude rejection step after baseline correction. ICA with MARA was retained because it has been validated for robust artifact removal across multiple EEG paradigms, including ocular and muscle artifacts^43,44^. Signals were re-referenced, filtered, segmented, and subjected to multi-step artifact rejection (including ICA-based removal) before channel interpolation and baseline correction. Participants were required to retain at least 30% of both total and infrequent target trials (142 in total), and subjects not meeting these criteria were excluded prior to all analyses and were not reported as part of our study sample. Across all included participants, an average of 133 infrequent trials were retained (median = 136, range = 73–142). Detailed thresholds for epoch/channel exclusion and other preprocessing steps are described in the Supplemental Methods.

For P3b analysis, ERPs of all the infrequent target trials were averaged at each electrode. Region averages were computed for three regions: Frontal (“FP1”, “FP2”, “F3”, “F4”, “Fz”, “F7”, “F8”, “FC1”, “FC2”, “FC5”, “FC6”), Central (“C3”, “C4”, “Cz”), and Parietal (“P3”, “P4”, “Pz”). Regions were defined to enhance interpretability, while results for midline electrodes (“Fz”, “Cz”, “Pz”) were separately reported in the Supplemental Results. The averaged ERPs were subsequently bandpass filtered (0.05–30 Hz, a zero-phase IIR filter using MNE-Python^45^). In this approach, a broader filter (0.05–70 Hz) was first applied prior to ICA to suppress high-frequency noise, while the final 0.05–30 Hz filter restricted analyses to the conventional ERP frequency range. For P3b component analysis, latency and amplitude were extracted from the filtered averaged ERP waveforms at each cluster. Peaks were identified within the 250–500 ms window using a positive peak detection algorithm (get_peak) from MNE-Python^45^, capturing the latency and amplitude of the most positive value within the specified time window. Amplitude and latencies for the target tone were used for further analysis.

### MRI acquisition and preprocessing

All participants were scanned on a Siemens 3 T Magnetom Prisma scanner at the Neuroimaging Core Unit Munich (NICUM) using a 32-channel head coil. T1-weighted images were acquired with a magnetization-prepared rapid acquisition gradient echo sequence (slice thickness: 0.8 mm, repetition time: 2500 ms, echo time: 2.22 ms, flip angle: 8°, FoV: 256 mm^2^).

For single-voxel proton magnetic resonance spectroscopy (¹H-MRS), trained MRI operators manually placed a 20 × 20 × 20 mm^3^ volume of interest (VOI) in the anterior cingulate cortex (ACC; for 56 HC and 58 SSD participants) or a 30 ×30 x 15 mm^3^ VOI in the left dorsolateral prefrontal cortex (lDLPFC; for 51 HC and 49 SSD participants), using the T1-w images for anatomical guidance. For both regions, VOI placement used a template reference image and defined anatomical landmarks. Each participant contributed data from only one voxel location. A fastestmap sequence was used for B0 shimming, followed by a MEGA-semi-localized by adiabatic selective refocusing (MEGA-sLASER) sequence^46^ for MRS acquisition. MEGA-sLASER editing was performed with interleaved editing pulses at 1.90 ppm (ON) and 7.50 ppm (OFF) (flip angle = 180°, bandwidth = 100 Hz, echo time [TE] = 68 ms, repetition time [TR] = 3000 ms). This sequence is optimized for the detection of low-concentration metabolites like GABA. The sLASER component provides superior localization and reduces chemical shift displacement error compared to PRESS, while MEGA-editing effectively isolates the GABA signal from the overlapping creatine signal^47^. The 68 ms TE was chosen to maximize the efficiency of the editing process for the GABA signal^48,49^. For quantification of high-concentration metabolites like glutamate and Glx (a composite of glutamate and glutamine), the unedited OFF spectrum was used. The MEGA-PRESS OFF spectrum is functionally equivalent to a standard separately acquired PRESS spectrum and is a well-validated methodology for simultaneous glutamate or Glx and GABA measurements^50^. Each scan included a water-suppressed acquisition (128 averages) and a non-water-suppressed acquisition (8 averages), used as a reference to correct the baseline and improve background noise estimation.

MRS data were processed by using Osprey (v2.5.0)^51^ and LCModel (Linear Combination Model, v6.3-1R)^52^. Motion and frequency drift were corrected during preprocessing in Osprey through eddy-current correction, robust spectral registration, and rejection of motion-affected averages. Voxel placement consistency was checked visually using the generated coregistration images. All processed spectra were subsequently analyzed in LCModel, using basis sets specifically tailored for this sequence at the NICUM Siemens 3 T Magnetom Prisma scanner. These basis sets were generated through numerical simulation using the FID-A toolkit^53^ in MATLAB, matching the exact parameters of our custom MEGA-sLASER protocol, including TE = 68 ms, TR = 3000 ms, the durations and shapes of the RF pulses, and the frequencies of the ON (1.90 ppm) and OFF (7.50 ppm) editing pulses, to ensure the highest fidelity between simulated and in vivo spectra. Glx was derived from the OFF spectrum, and GABA from the DIFF spectrum. Finally, metabolite concentrations (millimolar, mM) were corrected for cerebrospinal fluid (CSF) contribution within the volume of interest (VOI) using individual T1w MRI segmentation values derived from the Osprey analysis.

### Statistical analysis

Analyses were guided by a stepwise pipeline: (1) identification of P3b disturbances between groups, (2) testing whether these disturbances were associated with metabolite levels, and (3) linking these associations to behavioral and cognitive measures. All statistical analyses were conducted using R (v4.4.3), with all tests two-sided. Age and sex were included as covariates in all analyses, and 95% confidence intervals (CI) were reported for all estimates. Missing data were treated as NA and not imputed. Hypothesis-driven tests (i.e., P3b reductions in patients and greater P3b reductions in more symptomatic patients) were corrected using false discovery rate (FDR^54^; with thresholds of *p*_*FDR*_ < .05), while uncorrected thresholds of *p* < .05 were applied to exploratory, post hoc analyses linking metabolite levels with P3b and cognition.

In total, the analytical pipeline comprised ANCOVAs for behavioral (reaction time and percentage of correct responses), ERP measures (three regions × two measures for both group- and cluster-based models), and metabolite comparisons (two regions × two metabolites). Regression analyses were conducted in two stages: (i) interaction models testing metabolite × group/cluster effects (group-based: central and parietal amplitudes; cluster-based: frontal, central, and parietal amplitudes), and (ii) post hoc within-group/cluster models limited to significant interactions.

Metabolite concentration values were subjected to quality control measures. Values with LCModel estimated standard deviations (SD%) based on Cramer-Rao lower bounds (CRLB) exceeding 30% or signal-to-noise ratios (SNR) less than 5 were replaced with missing values (NA). Subsequently, major outliers, values falling more than three times the interquartile range below the first quartile or above the third quartile, were identified^55^ and also replaced with NA.

All group differences were assessed using analysis of covariance (ANCOVA). For comparisons between two groups (e.g., SSD vs. HC), ANCOVA models included group as the between-subjects factor and age and sex as covariates. For comparisons involving three groups (HC, SSD−, SSD+), multilevel group ANCOVAs were performed. Post hoc pairwise comparisons were conducted using estimated marginal means (EMMs) contrasts. For hypothesis-driven ANCOVA tests, FDR correction was applied across regions and P3b measures, as well as within each model for post hoc contrasts. Cohen’s d was used as an estimate of the effect size for these contrasts, calculated based on EMMs and the residual standard deviation from the ANCOVA models.

Linear regression analyses were used to explore the relationships between metabolite levels and P3b measures. Models included P3b measures as the dependent variable and metabolite levels as predictors, along with the interaction between metabolite levels and group. Age and sex were included as covariates. To obtain robust estimates of the regression coefficients and associated p-values, bootstrapping methods were employed. Specifically, 10000 bootstrap samples were generated for each model using residual resampling. 95% confidence intervals were computed from the bootstrap distributions, and p-values were calculated by inverting the confidence intervals using boot.pval library (v0.7.0) in R^56,57^.

Partial correlation analyses were conducted to examine the relationships of metabolite levels and P3b measures with behavioral and cognitive measures, while controlling for age and sex. This method allowed for the assessment of the strength of associations between variables independent of the covariates.

To capture the symptom heterogeneity underlying P3b amplitude fluctuations within the patient group, k-means clustering (100 runs with random starting points, selecting the solution with minimal within-cluster variances) was performed based on PANSS total scores. Based on multiple clustering solutions evaluated with silhouette scores, a two-cluster solution was selected as the optimal solution (silhouette score = 0.62). This choice also reflects our aim to form the most clinically distinct groups characterized by symptom severity, while maintaining reasonable group sizes. Participants were thus assigned to one of two clusters: SSD− (lower-symptom) or SSD+ (higher-symptom), which were used in subsequent analyses.

**Fig. 1:**
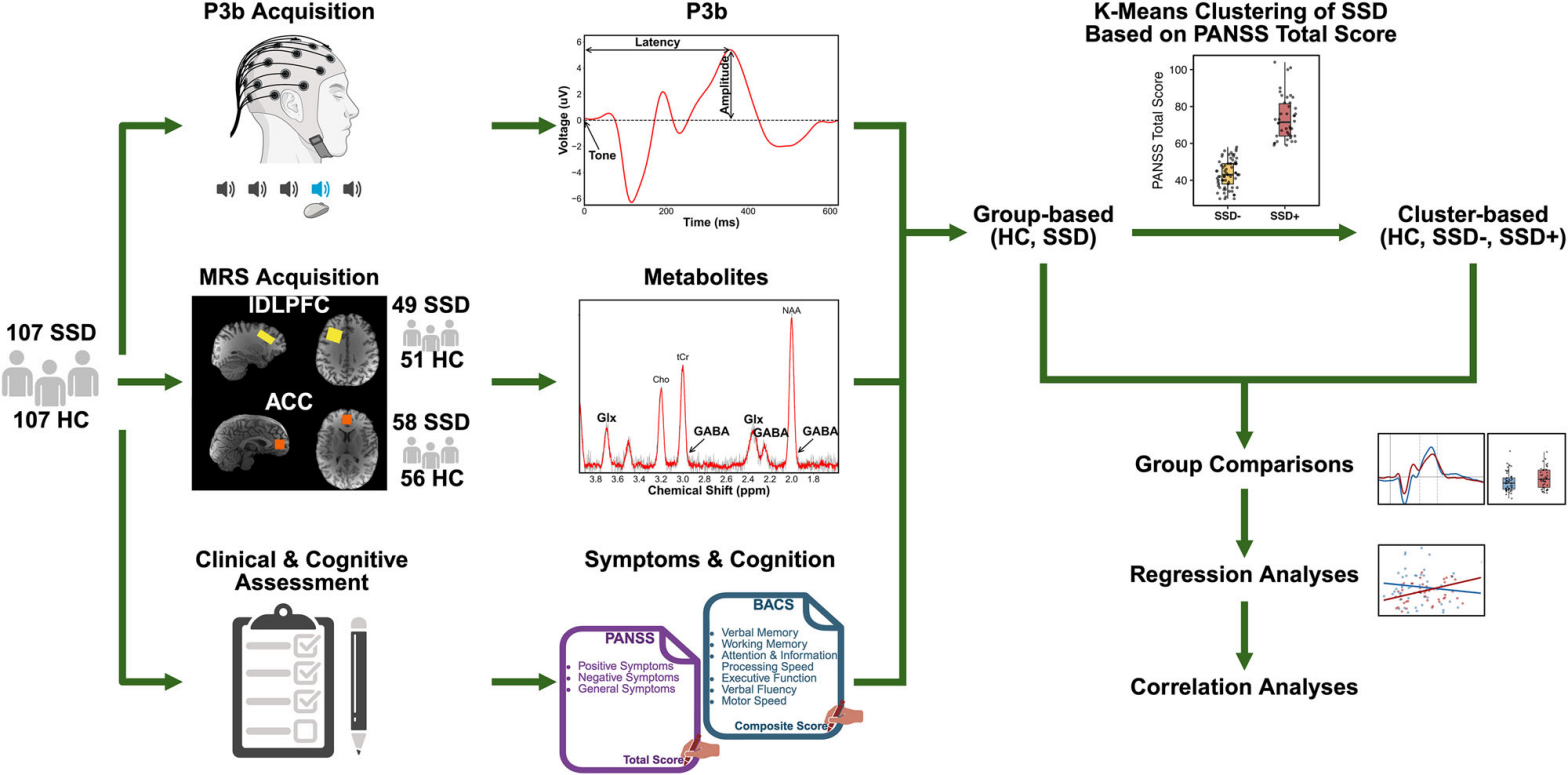
A summary diagram of the data acquisition and analysis. HC healthy controls, SSD schizophrenia spectrum disorder, SSD+ higher-symptom cluster, SSD− lower-symptom cluster, ACC anterior cingulate cortex, lDLPFC left dorsolateral prefrontal cortex, P3b positive peak around 300 ms in response to the target tone during the auditory oddball task, GABA gamma-aminobutyric acid, Glx glutamate-glutamine peak, BACS Brief Assessment of Cognition in Schizophrenia, PANSS The Positive and Negative Syndrome Scale.

For a summary of acquisition and analysis steps, please refer to Fig. 1.

## Results

### Clustering by PANSS scores

To address heterogeneity within our SSD sample, we clustered patients into two subgroups based on PANSS total scores, resulting in three clusters for further analysis: HC, SSD− (lower-symptom, *N* = 65), and SSD+ (higher-symptom, *N* = 41). One patient was not included in the clusters because PANSS scores were not available. We included both group-based (HC and SSD) and cluster-based analyses (HC, SSD−, and SSD+) for all the analyses. The SSD− cluster had significantly lower PANSS positive (Contrast EMM = −6.63, 95% CI [−7.81, −5.44], *d* = 2.23, *p* < 0.001), PANSS negative (Contrast EMM = −8.70, 95% CI [−9.79, −7.61], *d* = 3.18, *p* < 0.001), and PANSS total scores (Contrast EMM = −29.69, 95% CI [−32.43, −26.95], *d* = 4.31, *p* < 0.001), reflecting fewer symptoms. Additionally, the SSD− cluster exhibited higher BACS composite z-scores (Contrast EMM = 0.90, 95% CI [0.38, 1.42], *d* = 0.71, *p* < 0.001), indicating better cognitive function. In summary, the SSD− cluster presented with fewer symptoms and better cognitive function compared to the SSD+ cluster, as indicated by lower PANSS scores and higher BACS composite z-scores.

### Behavioral and ERP measures for P3b

The SSD group demonstrated slower reaction times (RTs) than the HC group (*F*(1, 210) = 16.75, *p* < 0.001; mean HC = 339 ms, mean SSD = 386 ms) and a lower percentage of correct responses (*F*(1, 210) = 7.47, *p* = 0.007; mean HC = 98.6%, mean SSD = 95.7%) for the auditory oddball task.

**Fig. 2:**
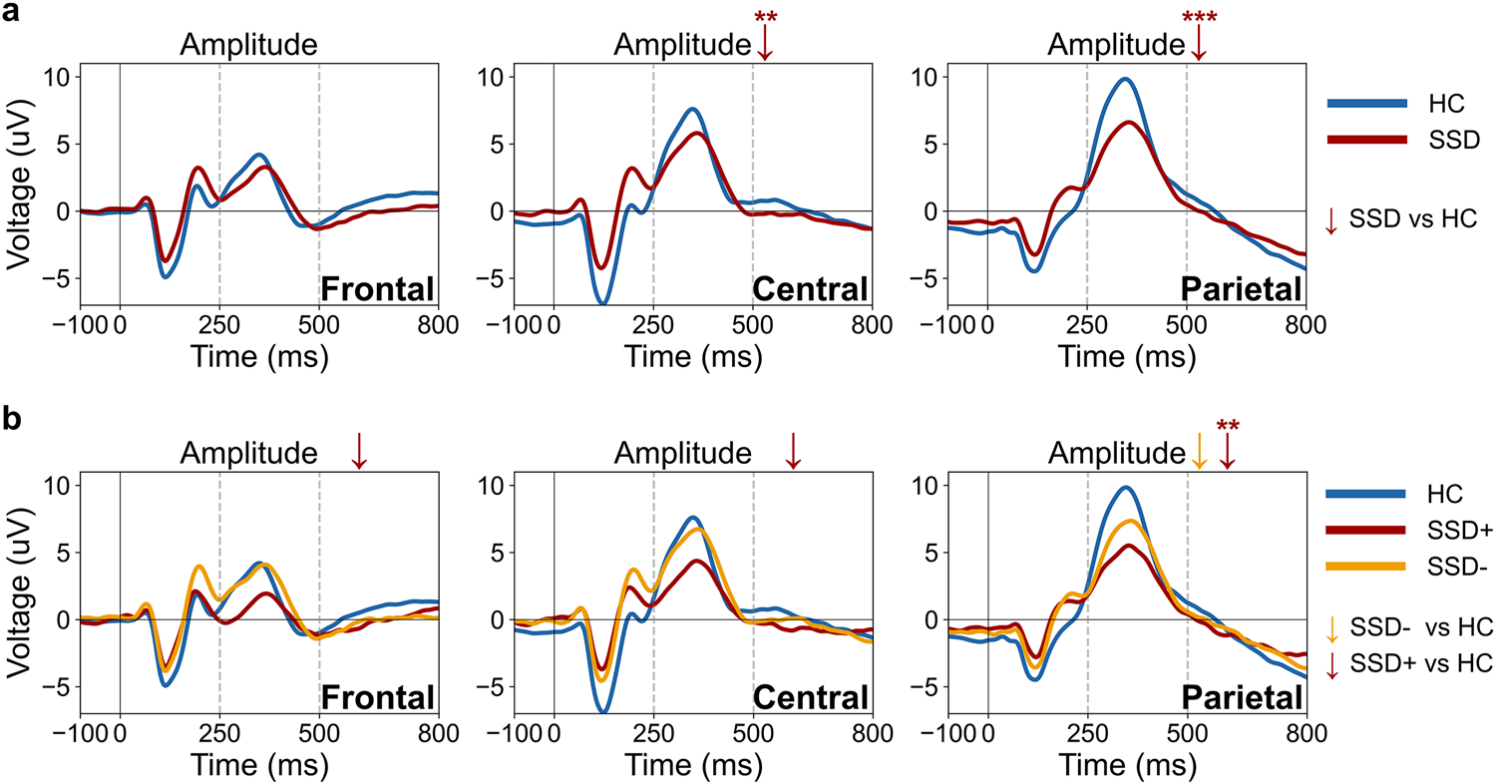
Frontal, central, and parietal ERPs. P3b amplitude and latency differences across: **a** HC and SSD groups, and **b** HC, SSD−, and SSD+ clusters. The red (SSD, SSD+) and yellow (SSD−) arrows (↓) show the decrease in amplitude compared to HC, with arrows without asterisks indicating trend-level effects (p < 0.1). HC healthy controls, SSD schizophrenia spectrum disorder, SSD− lower-symptom cluster, SSD+ higher-symptom cluster, ms milliseconds, μV microvolts. Significance level (FDR): *< 0.05, **< 0.01, ***<0.001. See Table 2 for group means, standard deviations, and sample sizes for each measure.

Consistent with these behavioral impairments, the SSD group also exhibited decreased P3b amplitudes at central (*F*(1,206) = 9.52, *p* = .002, *p*_*FDR*_ = .007) and parietal (*F*(1,209) = 29.22, *p* < .001, *p*_*FDR*_ < 0.001) electrode sites (see Fig. 2a). Frontal P3b amplitude did not differ significantly between groups (*F*(1,199) = 3.28, *p* = 0.072, *p*_*FDR*_ = 0.144). No significant differences were observed in P3b latency across all regions (all *p*s > .05).

P3b amplitude comparisons of clusters further highlighted distinct electrophysiological profiles among the clusters. The omnibus ANCOVAs revealed significant group effects at frontal (*F*(2,197) = 4.66, *p* = 0.011, *p*_*FDR*_ = 0.021), central (*F*(2,204) = 6.52, *p* = 0.002, *p*_*FDR*_ = 0.005), and parietal sites (*F*(2,207) = 15.63, *p* < 0.001, *p*_*FDR*_ < 0.001). Post-hoc contrasts showed that the SSD+ cluster (higher-symptom) had significantly reduced amplitudes at parietal (Contrast EMM = −2.91, 95% CI [ − 4.66, −1.15], *d* = 0.62, *p* = 0.001, *p*_*FDR*_ = 0.004), with trend-level amplitude reductions at central (Contrast EMM = −2.08, 95% CI [ − 3.96, −0.20], *d* = 0.42, *p* = 0.031, *p*_*FDR*_ = 0.092) and frontal (Contrast EMM = −1.58, 95% CI [−3.05, −0.12], *d* = 0.41, *p* = 0.035, *p*_*FDR*_ = 0.075) sites after FDR correction (see Fig. 2b). In contrast, the SSD− cluster (lower-symptom) showed a trend-level amplitude reduction compared to HC only in the parietal region (Contrast EMM = −1.54, 95% CI [−3.05, −0.03], *d* = 0.33, *p* = 0.046, *p*_*FDR*_ = 0.069) (see Fig. 2b). Additionally, there was a trend-level reduction in frontal amplitudes in SSD+ compared to SSD− (Contrast EMM = 1.56, 95% CI [ − 3.121, 0.001], *d* = 0.41, *p* = 0.050, *p*_*FDR*_ = 0.075). No significant differences were observed in latencies for any comparison. Additionally, there were no significant behavioral differences during the task between the SSD− and SSD+ clusters in terms of RTs or percentage of correct responses (all *p*s > 0.05).

### Metabolite comparisons

**Fig. 3:**
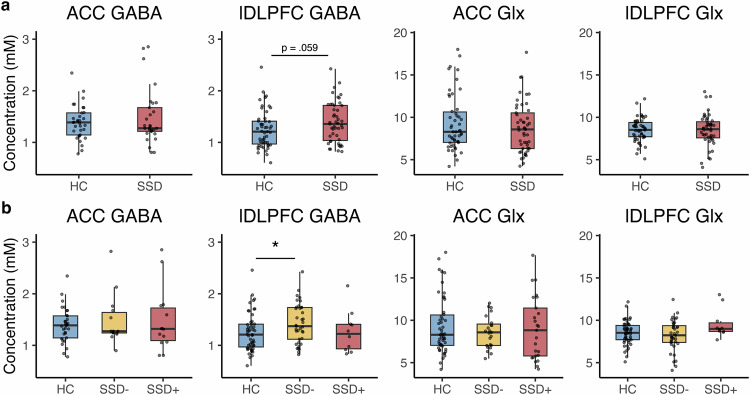
ACC and lDLPFC GABA and Glx levels. Metabolite levels across: **a** HC and SSD groups, and **b** HC, SSD−, and SSD+ clusters. HC healthy controls, SSD schizophrenia spectrum disorder, SSD− lower-symptom cluster, SSD+ higher-symptom cluster, ACC anterior cingulate cortex, lDLPFC left dorsolateral prefrontal cortex, GABA gamma-aminobutyric acid, Glx glutamate-glutamine peak, mM millimolar. Box-plot elements are defined as follows: the center line represents the median, the box limits indicate the upper and lower quartiles (25th and 75th percentiles), and the whiskers extend to 1.5 times the interquartile range (IQR). Each point represents individual subject data. Significance level: *< 0.05, **< 0.01, ***<0.001. See Table 2 for group means, standard deviations, and sample sizes for each measure.

No significant group effects were observed for GABA or Glx in the ACC, nor for Glx in the lDLPFC (all *p*s > 0.05) when comparing HC and overall SSD, although there was a trend toward higher lDLPFC GABA levels in patients, it did not reach statistical significance (*F*(1, 94) = 3.65, *p* = 0.059; mean HC = 1.25, mean SSD = 1.39) (see Fig. 3a).

An ANCOVA with the three clusters (HC, SSD− [lower-symptom], SSD+ [higher-symptom]) indicated a significant cluster effect for lDLPFC Glx levels (*F*(2, 94) = 3.42, *p* = 0.037) and a trend-level cluster effect for lDLPFC GABA levels (*F*(2, 93) = 2.63, *p* = 0.078). Due to the small sample size of SSD+ subjects with lDLPFC metabolite data (*N* = 10), which could introduce bias from unequal cluster sizes^58^, we conducted an additional ANCOVA including only the HC and SSD− clusters. This follow-up analysis revealed a significant increase in lDLPFC GABA levels in the SSD− cluster compared to HC (*F*(1, 84) = 5.15, *p* = 0.026), while lDLPFC Glx levels did not significantly differ between the SSD− and HC clusters (*F*(1, 85) = 1.50, *p* = 0.225) (see Fig. 3b). No significant metabolite differences were observed in the ACC across clusters.

### Follow-up analysis: group-based (overall SSD and HC) regression and correlation analyses

**Fig. 4:**
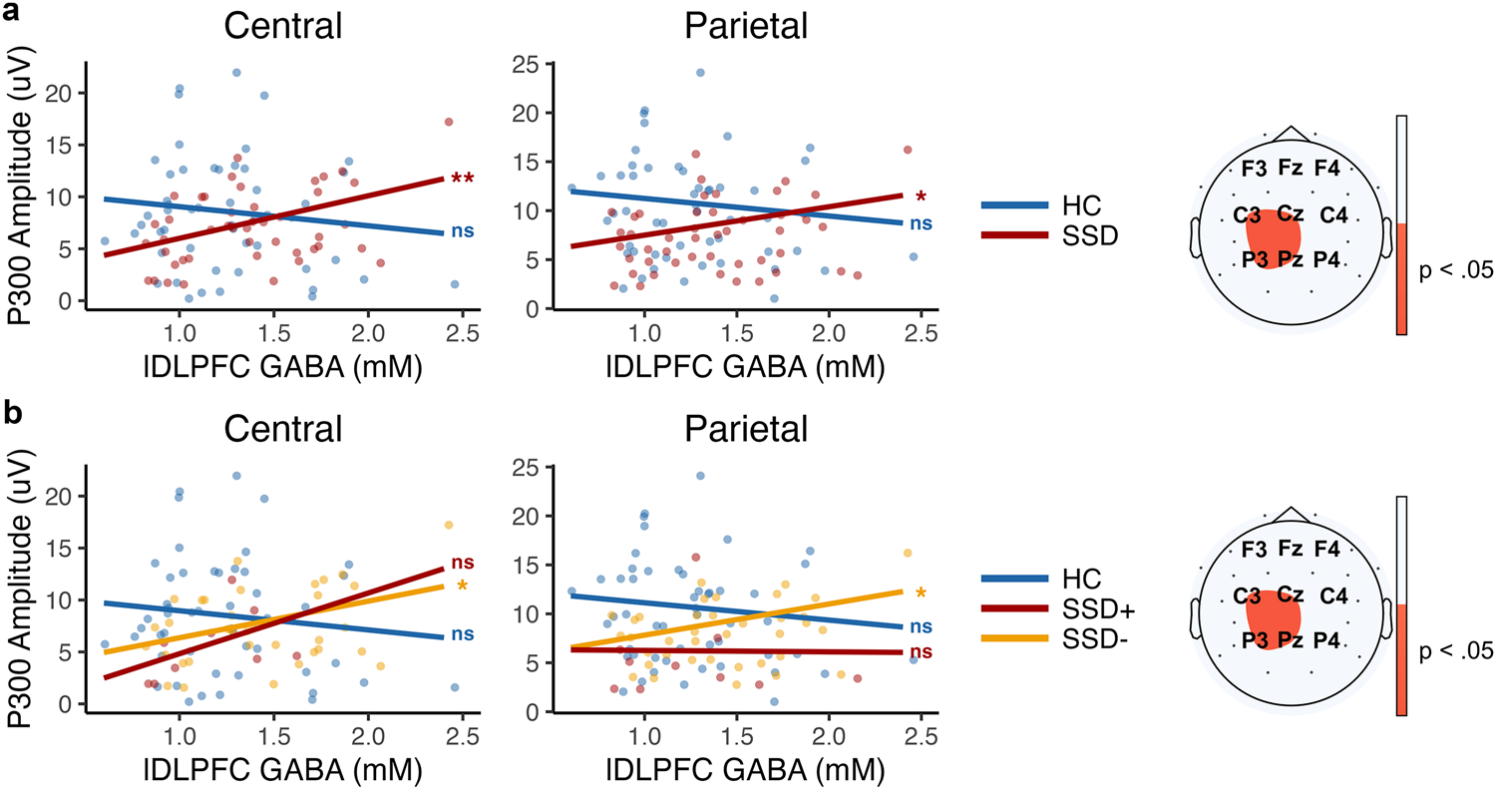
Central and parietal P3b amplitudes and GABA relationship. P3b and GABA relationship on the left, and significant electrodes for these relationships on the right across: **a** HC and SSD groups, and **b** HC, SSD−, and SSD+ clusters. HC healthy controls, SSD schizophrenia spectrum disorder, SSD− lower-symptom cluster, SSD+ higher-symptom cluster, μV microvolts, mM millimolar. Central electrodes: C3, Cz, C4. Parietal electrodes: P3, Pz, P4. Electrodes are placed according to the international 10–20 system. Significance level: *<0.05, **<0.01, ***<0.001, ns not significant. See Supplementary Table 5 for detailed sample sizes used in each analysis.

To explore whether metabolite levels could account for the P3b amplitude differences observed between groups at central and parietal electrodes, we conducted linear regression analyses. Significant lDLPFC GABA × Group interactions were observed at both central (*B* = 5.94, 95% CI [0.99, 10.88], *p* = 0.020) and parietal sites (*B* = 4.69, 95% CI [0.04, 9.40], *p* = 0.048). Follow-up within-group regressions showed no significant associations in HC (*p*s > 0.05), but robust positive associations in SSD (central: *B* = 4.42, 95% CI [1.70, 7.10], *p* = 0.001; parietal: *B* = 3.08, 95% CI [0.44, 5.66], *p* = 0.020) (see Fig. 4a). However, GABA levels in the ACC were not associated with any P3b amplitudes, and Glx levels in either the lDLPFC or ACC did not demonstrate any significant relationship with P3b amplitudes. Given that the DLPFC ROI was in the left hemisphere, we also tested hemisphere-specific associations. Significant lDLPFC GABA × Group effects emerged at left (C3, P3) and midline (Cz, Pz), but not right (C4, P4), electrodes, with effects limited to the SSD group (see Supplemental Results).

To link these regional associations to behavior and cognition in the SSD group, we correlated lDLPFC GABA levels and P3b amplitudes at central and parietal sites with RTs, percentage of correct responses, and the BACS composite z-scores. Central and parietal P3b amplitudes were positively correlated with percentage of correct responses (central: *r* = 0.29, 95% CI [0.10, 0.45], *p* = 0.003; parietal: *r* = 0.35, 95% CI [0.17, 0.50], *p* < 0.001), while only parietal P3b amplitude showed a positive correlation with the BACS composite z-score (*r* = 0.21, 95% CI [0.01, 0.39], *p* = 0.039) and a negative correlation with RTs (*r* = −0.27, 95% CI [ − 0.44, −0.08], *p* = 0.006). Notably, GABA levels were not correlated with any of the behavioral or cognitive measures (all *p*s > .05).

### Follow-up analysis: cluster-based (SSD− and SSD + ) regression and correlation analyses

We repeated the regression analyses in the cluster-based groups (HC, SSD− [lower-symptom], SSD+ [higher-symptom]). Significant lDLPFC GABA × Cluster interactions were observed at both central (*B* = 5.38, 95% CI [0.16, 10.66], *p* = 0.043) and parietal sites (*B* = 4.95, 95% CI [0.06, 9.95], *p* = 0.046). Follow-up within-group regressions showed only significant positive associations in SSD− cluster (central: *B* = 3.65, 95% CI [0.52, 6.77], *p* = 0.024; parietal: *B* = 3.05, 95% CI [0.24, 5.91], *p* = 0.031) (see Fig. 4b). Consistent with prior analyses, no significant relationships were found in models involving GABA levels in the ACC or Glx levels in the lDLPFC or ACC (all *p*s > 0.05). Exploratory electrode-wise analyses further showed that lDLPFC GABA × Cluster interactions were significant at left (C3, P3) and midline (Cz, Pz) electrodes, but not at right (C4, P4), with effects limited to the SSD− cluster (see Supplemental Results).

To link these associations to behavior and cognition in the SSD− cluster, we performed correlation analyses between GABA levels in the lDLPFC and P3b amplitudes at central and parietal sites with RTs, percentage of correct responses, and the BACS composite z-scores. Central and parietal P3b amplitudes were positively correlated with percentage of correct responses (central: *r* = 0.29, 95% CI [0.05, 0.50], *p* = 0.021; parietal: *r* = 0.37, 95% CI [0.13, 0.56], *p* = 0.003), while only parietal P3b amplitude showed a negative correlation with RTs (*r* = −0.26, 95% CI [−0.47, −0.01], *p* = 0.043). No correlations were found between central P3b amplitudes and the BACS composite z-score. Similar to the SSD group, GABA levels were not correlated with any of the behavioral or cognitive measures in this cluster (all *p*s > 0.05).

## Discussion

This study investigated the potential role of GABA and Glx levels in the ACC and lDLPFC in relation to P3b ERP differences in SSD, and their links to cognition and behavior. Clustering the SSD group into higher (SSD+) and lower (SSD−) symptom subgroups provides insight into the heterogeneous nature of GABAergic function and its impact on cognitive processes across different symptom profiles.

Analysis of P3b amplitudes revealed significant differences between the SSD and HC groups, with reduced amplitudes in the parietal and central regions in the SSD group. Subgroup analysis revealed that higher-symptom patients showed significantly reduced P3b amplitudes at parietal sites, with trend-level reductions at central and frontal regions. In contrast, lower-symptom patients, with fewer symptoms and better cognitive function, showed a trend-level reduction in P3b amplitude only in the parietal region. Regarding metabolites, lower-symptom patients had higher GABA levels in the lDLPFC compared to HC, whereas no significant differences were found in the overall SSD group. Higher GABA levels in the lDLPFC were associated with increased P3b amplitudes at central and parietal sites for the overall SSD and lower-symptom groups. Although lDLPFC GABA levels were linked to P3b amplitudes, which in turn positively correlated with the BACS composite score and behavioral performance, GABA itself did not directly correlate with behavioral or cognitive measures indicating rather indirect impact on the neurophysiology and cognition outcome in this cohort.

### Amplitude differences across regions

P3b amplitude comparisons revealed distinct ERP profiles between patients and healthy controls, with higher and lower-symptom patients showing different patterns of impairment. P3b amplitude was significantly reduced in the SSD group compared to HC in the parietal and central regions, aligning with the well-replicated amplitude reductions in SSD^3,4^. Higher-symptom patients exhibited significantly lower P3b amplitudes than healthy controls at parietal sites, with trend-level reductions at central and frontal regions, suggesting widespread but predominantly parietal impairment. In contrast, lower-symptom patients showed a trend-level amplitude reduction compared to controls only in the parietal region, with preserved frontal and central amplitudes. This pattern aligns with the literature showing that the degree of P3b reduction varies with symptom severity^10,11^. Correlations between P3b amplitude and behavioral and cognitive performance linked higher amplitudes to better cognitive performance, consistent with previous research demonstrating the relationship between P300 and various cognitive domains (see Hamilton et al.^5^ for a review). Additionally, lower-symptom patients had higher BACS composite scores than higher-symptom patients, further highlighting the relationship between cognitive impairment and symptom severity. While P3b amplitudes correlated with cognitive measures, GABA levels showed no direct associations, suggesting that P3b may be a more immediate marker of cognitive function, with GABA’s influence potentially being indirect or mediated by other factors.

### GABAergic dysfunction in the ACC and lDLPFC

We observed increased GABA levels in the lDLPFC specifically for lower-symptom patients but not for the broader SSD patient group. Kegeles et al.^59^ found no changes in lDLPFC GABA levels compared to controls, likely reflecting differences in symptom severity and sample composition, whereas postmortem studies consistently report reduced GAD67 alongside broader GABAergic abnormalities in the DLPFC, including altered GABA_A_ receptor binding, GAD65, and GAT-1 (see De Jonge et al.^60^ for a review). While our in vivo finding of increased lDLPFC GABA in lower-symptom patients does not directly correspond to the GAD67 reductions, it can be interpreted within this broader evidence of GABAergic dysfunction in the DLPFC.

Furthermore, we found a positive association between lDLPFC GABA levels and P3b amplitude within the patient group, particularly among the lower-symptom subgroup. While no previous studies have directly linked MRS-measured GABA to P300, our findings differ from studies on GABA agonists in healthy individuals, showing a negative GABA and P300 amplitude association^61–63^. This distinction may highlight the differences between acute GABA modulation in healthy subjects and chronic GABA alterations observed in SSD. Unlike acute GABA increases in healthy subjects, which can temporarily suppress cortical activity, chronically elevated GABA in SSD may reflect a compensatory mechanism restoring the E/I balance. Additionally, the positive relationship in our patients, absent in controls, highlights how GABA-related modulation of P3b may reflect the distinct neuroadaptive processes in SSD.

Our findings support prior studies highlighting the DLPFC’s role in SSD pathophysiology, particularly in cognitive domains such as working memory and cognitive control^21^. The DLPFC’s involvement in these domains makes it particularly sensitive to shifts in the excitatory-inhibitory (E/I) balance^64^, with a potential GABAergic compensation mechanism buffering against such shifts. Previous studies suggest that GABAergic dysfunction affects the DLPFC’s capacity to regulate excitatory inputs, thereby supporting cognitive functions^65^. Increased GABA levels in our lower-symptom patients could represent an adaptive mechanism to stabilize dysfunction and maintain cognitive control, as suggested by more preserved P3b amplitudes and cognitive performance in this group. In contrast, the higher-symptom group may lack sufficient GABAergic compensation, leading to broader cognitive deficits, where increased inhibition alone no longer stabilizes the E/I balance.

In contrast to the lDLPFC, ACC GABA levels did not differ from healthy controls in patients or subgroups, consistent with recent meta-analyses reporting no significant ACC GABA differences in schizophrenia^25,29^. Some prior MRS studies have shown increased GABA levels in the ACC for ultra-high-risk (UHR) individuals, patients with first-episode psychosis (FEP), and medication-naïve patients with schizophrenia compared to healthy controls^59,66–69^, highlighting the role of GABAergic dysfunction or compensation in early stages of illness or in medication-naïve conditions. However, our sample consisted of mostly chronic patients receiving antipsychotic treatment. A study with a chronic schizophrenia sample reported increased ACC GABA/Cr ratios; however, creatine (Cr) as an internal reference has been questioned due to its potential fluctuations in illness states^70^. Furthermore, in the study of Kegeles et al.^59^, although ACC GABA was increased in the medication-free group, the medication-receiving group did not show any differences with healthy controls. Similarly, an intervention study on FEP patients found that ACC GABA elevations present at baseline disappeared after four weeks of antipsychotic treatment^71^ likely due to medication normalization. Together, these findings and study population differences suggest that antipsychotic treatment and longer duration of the illness may stabilize or normalize GABA levels in the ACC, potentially accounting for the lack of significant changes observed in our sample.

Overall, our findings suggest a potential region-specific compensatory mechanism in GABA functioning. This mechanism may optimize the excitation-inhibition balance, enhancing cognitive processing in less symptomatic patients. However, this compensatory mechanism may break down with illness progression or higher symptom severity, as seen in our higher-symptom group. While acute increases in GABAergic inhibition are likely to suppress cortical activity, in SSD, persistent region-specific GABA elevations might stabilize the E/I balance over time, supporting cognitive functioning.

### Glx levels and the role of glutamatergic dysfunction

Unlike GABA, Glx was not linked to P3b amplitude and showed no significant differences between SSD and HC in either the ACC or lDLPFC. Although studies report reductions in glutamate concentrations within medial frontal regions, these findings are accompanied by increased interindividual variability, particularly among older patients or with greater symptom severity^26–28^. Prior work in healthy samples, investigating the associations between glutamatergic and P300 measures, found that ACC Gln/Glu ratio and Gln, but not Glu or Glx, were associated with frontal P3a amplitude, while no associations between parietal–occipital cortical glutamatergic measures and parietal P3b amplitude were observed^34^. Consistent with Hall et al.^34^, our study, which focused on P3b amplitude, also found no associations between Glx and P3b in the ACC or lDLPFC. The absence of Glx group differences and Glx-P3b amplitude relationships in our study does not rule out the relevance of glutamatergic dysfunction and its link to cognition in SSD. MRS primarily captures intracellular glutamate levels and cannot directly assess synaptic transmission or excitatory signaling^72,73^. Bojesen et al.^74^ further suggest that cognitive deficits in schizophrenia may be linked to impaired glutamate dynamics during cognitive tasks rather than at resting levels. Additionally, NMDAR hypofunction has been implicated in cognitive deficits and P300 abnormalities in schizophrenia^5^. The role of glutamate-glutamine in task-specific contexts or in receptor-mediated processes highlights a direction for further research on SSD-related cognitive deficits.

### Limitations, strengths, and future directions

This study has several limitations. First, medication effects remain a concern, and although we did not find any relationships between GABA levels or P3b amplitudes and chlorpromazine equivalent dose (CPZeq, see the Supplemental Results), this measure does not capture differences in drug classes, and its longitudinal changes remain unknown in this study. Second, the smaller SSD+ group size limits statistical power, and while our overall sample size exceeds many MRS studies, it remains a constraint for detecting smaller effects. Third, the lack of simultaneous MRS-EEG recordings and the use of resting-state rather than task-based MRS limit the assessment of dynamic neurochemical-electrophysiological interactions. Fourth, P3b recordings were conducted under eyes-closed conditions, raising concerns about alpha contamination, although prior meta-analytic evidence indicates no systematic effect of eye condition on P3b amplitude or latency^75^. This choice ensured comparability with our spectroscopy recordings, which were likewise performed with eyes closed. Fifth, the small voxel sizes for edited MRS measurements raise concerns about spectral quality; however, group-wise means and standard deviations for spectral quality and metabolite fits indicated that values were within acceptable ranges despite some group differences (see Supplementary Table 4). Lastly, the cross-sectional design limits causal interpretations, highlighting the need for longitudinal research. Despite these limitations, this study benefits from a larger sample than most prior MRS studies, the integration of MRS, EEG, and cognitive measures, and a symptom-based subgroup analysis. Future research should address these limitations by incorporating larger, balanced samples, longitudinal designs, and simultaneous task-based MRS-EEG to capture dynamic metabolite-electrophysiology interactions, while also accounting for different drug classes and their longitudinal variations.

## Conclusion

These findings suggest that region-specific GABAergic alterations in the lDLPFC may be linked to differences in cognitive function and neurophysiology in SSD. The observed links between GABA, P3b, and cognitive performance emphasize the complex interplay of neurochemical and electrophysiological processes in SSD. The variation in results across symptom profiles emphasizes the importance of considering heterogeneity within SSD subgroups when interpreting neurochemical and cognitive findings. While increased GABA in the lDLPFC of lower-symptom patients may reflect an adaptive mechanism supporting cognitive function, the absence of similar findings in higher-symptom patients suggests a potential breakdown of this process with greater illness severity. However, this study was purely exploratory, and this suggested mechanism should be tested in a follow-up hypothesis-driven study while accounting for the limitations we reported. In contrast, no significant alterations in ACC GABA were observed, which may reflect stabilization effects of chronic illness or antipsychotic treatment. These findings highlight the importance of considering regional and symptom-based differences.

## Supplementary information

Supplemental Material

## Data Availability

Data and code used for analysis in this paper will be shared by the lead contact upon request.

## Code availability

Data and code used for analysis in this paper will be shared by the lead contact upon request
