# Supplementary material for: The link between GABA levels and P300 abnormalities in schizophrenia spectrum disorders: regional and symptom-based insights
Source: Schizophrenia (Heidelb). 2026 Jan 23;12(1):11. doi: 10.1038/s41537-026-00730-5 (PMC12859150; doi:10.1038/s41537-026-00730-5)
Supplement: Supplementary file 1 — Supplemental Material [file 41537_2026_730_MOESM1_ESM.docx]

# Supplemental Methods

#### CDP Working Group

Stephanie Behrens, Emanuel Boudriot, Man-Hsin Chang, Valéria de Almeida, Sylvia de Jonge, Fanny Dengl, Peter Falkai, Laura E. Fischer, Nadja Gabellini, Vanessa Gabriel, Sabrina Galinski, Thomas Geyer, Katharina Hanken, Alkomiet Hasan, Genc Hasanaj, Alexandra Hisch, Georgios Ioannou, Iris Jäger, Marcel S. Kallweit, Temmuz Karali, Susanne Karch, Berkhan Karslı, Daniel Keeser, Christoph Kern, Nicole L. Klimas, Maxim Korman, Nikolaos Koutsouleris, Lenka Krcmar, Verena Meisinger, Julian Melcher, Matin Mortazavi, Joanna Moussiopoulou, Karin Neumeier, Frank Padberg, Boris Papazov, Irina Papazova, Sergi Papiol, Pauline Pingen, Oliver Pogarell, Siegfried G. Priglinger, Florian J. Raabe, Lukas Roell, Moritz J. Rossner, Philipp Sämann, Andrea Schmitt, Susanne Schmölz, Eva C. Schulte, Enrico Schulz, Benedikt Schworm, Elias Wagner, Sven Wichert, Vladislav Yakimov, Peter Zill, Zhuanghua Shi, Michael J. Ziller

#### Clinical and Cognitive assessments

Both patients and healthy controls underwent comprehensive clinical assessments. Basic socio-demographic data, family and psychiatric history, suicidality, and lifetime substance use were collected using the Munich Mental Health Biobank self-report questionnaire. Patient diagnoses were confirmed using the MINI International Neuropsychiatric Interview (v7.0.2) based on DSM-V criteria, and symptom severity was assessed with the Positive and Negative Syndrome Scale (PANSS)^1^. Additional information on psychiatric history, demographics including sex assigned at birth, lifestyle factors, somatic conditions, body mass index (BMI), and medications was obtained through self-reports and medical records. The Global Assessment of Functioning (GAF) scale assessed participants’ general functioning. The Andreasen criteria were used to differentiate remission status^2^. Antipsychotic doses were converted to chlorpromazine equivalents (CPZeq) using the defined daily dose method^3^. Smoking status was assessed using the Fagerström Test for Nicotine Dependence^4^.

Neurocognitive function was assessed using the Brief Assessment of Cognition in Schizophrenia (BACS), a validated tool for evaluating cognitive deficits in individuals with schizophrenia^5^. The BACS, administered exclusively to participants with native-level German proficiency, consisted of seven tasks measuring six cognitive domains: verbal memory (list learning), working memory (digit sequencing), motor speed (token motor task), verbal fluency (category instances and controlled oral word association), attention and information processing speed (symbol coding), and executive function (Tower of London test)^6^. The assessment took approximately 40 minutes to complete. For data analysis, z-scores were calculated for each task using the mean and standard deviation of the HC group. These task z-scores were averaged into a single score, which was then transformed into the BACS composite score with a final z-score transformation based on the HC mean and standard deviation of the average score^5^.

#### P3b Preprocessing

Raw EEG data from the auditory oddball task were first converted to BIDS format using BV2BIDS (Brain Products, Martinsried, Germany). EEG signals were re-referenced to the left and right mastoids, downsampled to 256 Hz, bandpass filtered between 0.05 and 70 Hz, and notch filtered at 49.5–50.5 Hz using pop_eegfiltnew, which implements zero-phase FIR filtering. The data were then segmented into 1.5-second epochs (-500 ms to 1000 ms relative to stimulus onset). Artifact removal involved a multi-step approach: epochs exceeding ±5 SD, showing linear trends (max slope=5, min R2=0.7), or containing low (<2 Hz; power threshold -50 to 50 dB) or high (20–40 Hz; power threshold -100 to 25 dB) frequencies were excluded. Channels were evaluated for poor quality using power spectrum (-4 to 6 std), kurtosis (-7 to 15 std), and joint probability (-9 to 7 std) thresholds. When >50% of epochs were marked for rejection, priority was given to channel removal, followed by epoch rejection based on the initial data. Datasets were excluded if >50% of data or >20% of electrodes were rejected. Independent component analysis (ICA) was then performed, and artifact components were removed using MARA^7^. Finally, a second round of rejection with adjusted thresholds (power spectrum [-6 to 5 std], kurtosis [-6 to 9 std], and joint probability [-7 to 7 std]) was conducted, and interpolations were applied to the removed channels. Baseline correction was performed using a -100 to 0 ms prestimulus window, and epochs exceeding ±100 µV were excluded. Cleaned data were converted back to continuous format for further analysis.

# Supplemental Results

#### Midline Electrode P3b Comparisons

The SSD group exhibited decreased P3b amplitudes across all three midline electrodes: Fz (*F*(1,197) = 5.25, *p* = .023, *p_FDR_* = .046), Cz (*F*(1,203) = 6.96, *p* = .009, *p_FDR_* = .027), and Pz (*F*(1,209) = 26.13, *p* < .001, *p_FDR_* < .001) (see [Supplementary Figure 1](#_ywvm37n94e3d)a). No significant differences were observed in P3b latency across midline electrodes (all *p*s > .05).

P3b amplitude comparisons of clusters (HC, SSD− [lower-symptom], SSD+ [higher-symptom]) further highlighted distinct electrophysiological profiles among the clusters. Compared to the HC cluster, the SSD+ cluster had significantly lower P3b amplitudes at Pz (Contrast EMM = −2.96, 95% CI [−4.90, −1.02], d = 0.57, *p* = .003, *p_FDR_* = .009), with trend-level reductions at Fz (Contrast EMM = −2.24, 95% CI [−4.20, −0.28], d = 0.44, *p* = .025, *p_FDR_* = .076) (see [Supplementary Figure 1](#_ywvm37n94e3d)b). In contrast, the SSD− cluster showed a trend-level amplitude reduction compared to HC only in the Pz electrode (Contrast EMM = −1.64, 95% CI [−3.31, 0.03], d = 0.32, *p* = .055, *p_FDR_* = .082) (see [Supplementary Figure 1](#_ywvm37n94e3d)b). No significant differences were observed in latencies for any comparison.

#### Hemisphere-Specific Regression Analyses

To investigate hemisphere-specific associations given our DLPFC ROI was in the left hemisphere, we analyzed C3, Cz, C4, P3, Pz, and P4 electrodes separately. Significant interactions between GABA levels in the lDLPFC and group were observed for left (C3, P3) and midline (Cz, Pz) electrodes in the central and parietal regions, but not for right electrodes. The interaction between GABA levels in the lDLPFC and group significantly predicted amplitude for C3 (*B* = 5.79, 95% CI [1.14, 10.31], *p* = .016), Cz (*B* = 6.73, 95% CI [0.77, 12.58], *p* = .025), P3 (*B* = 5.15, 95% CI [0.76, 9.64], *p* = .022), and Pz (*B* = 5.55, 95% CI [0.30, 10.87], *p* = .040). The GABA levels in the lDLPFC significantly predicted amplitudes only in the SSD group (C3: *B* = 3.45, 95% CI [0.66, 6.26], *p* = .016; Cz: *B* = 5.08, 95% CI [1.91, 8.34], *p* = .002; P3: *B* = 3.46, 95% CI [0.87, 6.15], *p* = .009; Pz: *B* = 3.35, 95% CI [0.29, 6.33], *p* = .030). No significant interaction associations were observed for right hemisphere electrodes of central and parietal regions (C4, P4; ps > .05).

We repeated the same analysis for cluster-based groups (HC, SSD− [lower-symptom], SSD+ [higher-symptom]). As in the previous analysis, significant associations were observed only for left (C3, P3) and midline (Cz, Pz) electrodes, but not for right electrodes. The interaction between lDLPFC GABA levels and cluster significantly predicted amplitudes for C3 (*B* = 6.29, 95% CI [1.38, 11.16], *p* = .012), Cz (*B* = 6.10, 95% CI [-0.01, 12.25], *p* = .050), P3 (*B* = 5.48, 95% CI [0.69, 10.29], *p* = .026), and Pz (*B* = 5.94, 95% CI [0.21, 11.73], *p* = .043). These associations were significant only in the SSD− cluster (C3: *B* = 3.84, 95% CI [0.53, 7.10], *p* = .023; Cz: *B* = 4.21, 95% CI [0.53, 7.98], *p* = .024; P3: *B* = 3.43, 95% CI [0.53, 6.30], *p* = .017; Pz: *B* = 3.35, 95% CI [0.16, 6.43], *p* = .037). No significant associations were observed for right hemisphere electrodes of the central and parietal regions (C4, P4; *p*s > .05).

#### P3b Frequency-Domain Analyses

To complement the amplitude-based P3b analyses, we computed time-frequency power using Morlet wavelets (1-7 Hz, 30 steps; number of cycles scaled with frequency to fit the epoch length). Power estimates were baseline-corrected (log-ratio to the -100 to 0 ms baseline) and averaged in the P3b window (250-500 ms). Analyses focused on delta (1-4 Hz) and theta (4-7 Hz) bands, which are most strongly implicated in P300 generation and altered in SSD^8–10^.

ANCOVAs revealed significantly reduced delta and theta power in SSD compared to HC across frontal (delta: *F*(1,210) = 24.57, *p_FDR_* < .001; theta: *F*(1,210) = 35.00, *p_FDR_* < .001), central (delta: *F*(1,210) = 32.35, *p_FDR_* < .001; theta: *F*(1,210) = 41.36, *p_FDR_* < .001), and parietal regions (delta: *F*(1,210) = 44.46, *p_FDR_* < .001; theta: *F*(1,210) = 39.79, *p_FDR_* < .001), in line with previous reports^8,9^. Regression models showed significant interactions between lDLPFC Glx and group for frontal theta power (*B* = 0.04, 95% CI [0.006, 0.073], *p* = .022), such that higher Glx predicted increased frontal theta power only in SSD (*B* = 0.02, 95% CI [0.001, 0.045], *p* = .042). Together, these results indicate that frequency-domain measures did not account for the metabolite-P3b amplitude associations observed in the main analyses but instead showed an additional effect of lDLPFC Glx on frontal theta power.

#### Control Analysis: Smoking and CPZeq Effects on lDLPFC GABA Levels and P3b Amplitudes

We examined the correlation between CPZeq and lDLPFC GABA levels, as well as P3b amplitudes in the central and parietal regions, within the SSD group, including age and sex as covariates. No significant correlations were observed (*p*s > .05). To further evaluate potential subgroup differences in medication exposure, we also compared CPZeq dosages between SSD- (lower-symptom and SSD+ (higher-symptom) clusters. No significant differences were found (SSD-: 319.28 ± 238.59, N = 62; SSD+: 387.33 ± 260, N = 41; Welch’s t-test, *p* = .183).

We ran an ANCOVA with age and sex as covariates to compare P3b amplitudes and lDLPFC GABA levels based on smoking status (smoker vs. non-smoker) within the SSD group. Smoking significantly decreased P3b amplitudes in all three regions: frontal (*F*(1,96) = 9.36, *p* = .003), central (*F*(1,100) = 9.39, *p* = .003), and parietal (*F*(1,101) = 5.27, *p* = .024). However, lDLPFC GABA levels did not differ significantly based on smoking status (*p* > .05).

To assess whether smoking status influenced our findings, we repeated the regression analyses including smoking as a covariate. For the group-based analyses, significant lDLPFC GABA × Group interactions were observed at both central (*B* = 6.00, 95% CI [1.13, 10.94], *p* = .014) and parietal sites (*B* = 4.65, 95% CI [0.01, 9.45], *p* = .049). Follow-up within-group regressions showed no significant effects in HC (*p*s > .05), but significant positive associations in SSD (central: *B* = 4.32, 95% CI [1.63, 7.08], *p* = .002; parietal: *B* = 3.05, 95% CI [0.48, 5.74], *p* = .020). For the cluster-based analyses, lDLPFC GABA × Cluster interactions were significant at both central (*B* = 5.43, 95% CI [0.19, 10.60], *p* = .041) and parietal sites (*B* = 4.96, 95% CI [0.03, 9.97], *p* = .048). Follow-up within-group cluster-based regressions revealed only significant positive associations in the SSD− cluster (central: *B* = 3.74, 95% CI [0.66, 6.72], *p* = .015; parietal: *B* = 3.13, 95% CI [0.29, 5.98], *p* = .029).

We found no significant associations between CPZeq and lDLPFC GABA levels or P3b amplitudes. Smoking was significantly associated with reduced P3b amplitudes, but controlling for smoking status did not affect the main findings of either the group-based or cluster-based analyses.

#### Control Analysis: Drowsiness/Sedation Effects Across Task Blocks

To address potential drowsiness and sedation effects, we divided the EEG signals into two blocks (first and last 9 minutes). Group comparisons were tested with a linear mixed-effects model including Group (HC, SSD), Region (frontal, central, parietal), Block, and their three-way and two-way interactions as fixed effects, with Age and Sex as covariates, and random intercepts for Region and Block within subject. Results showed a significant main effect of Group (*F*(1, 208.5) = 5.43, *p* = .021, *p_FDR_* = .027), with SSD group exhibiting reduced P3b amplitudes compared to HC. This replicates our main findings, confirming robust group differences. In addition, we observed a significant Group × Block interaction (*F*(1, 208.4) = 6.78, *p* = .010, *p_FDR_* = .015) and a Group × Region × Block interaction (*F*(2, 602.1) = 3.36, *p* = .035, *p_FDR_* = .040), indicating that group differences varied across task halves and regions. Follow-up contrasts showed that group differences were strongest in the first half (Central: *p* = .013; Parietal: *p* < .001), but weakened in the second half (Central: *p* = .323; Parietal: *p* < .001).

Even though group differences were robust, block interactions suggest a potential group-dependent influence of drowsiness or sedation. Therefore, we further tested whether the observed GABA-P3b amplitude associations at Central and Parietal sites in SSD could be explained by only block-related effects. For these associations, we fit a linear mixed-effects model of the form P3b Amplitude ~ GABA × Group + Block + Region + Age + Sex + (1|ID). Including Block as a covariate weakened the GABA × Group interaction to a nonsignificant trend (*F*(1, 97.9) = 3.09, *p* = .082). However, within-group analyses showed that in SSD group, GABA remained a significant positive predictor of P3b amplitude (*β* = 3.11, *SE* = 1.31, 95% CI [0.49, 5.74], *F*(1, 46.9) = 5.64, *p* = .022), whereas no effect was observed in HC (*p* = .420).

Together, these findings suggest that, although block-related effects were present, they did not fully account for the P3b amplitude group differences and GABA-P3b amplitude associations observed in SSD.

#### Control Analysis: GABA-P3b Amplitude Associations for Same Day EEG-Measurement

To ensure that our main findings were not influenced by the time interval between EEG and MRS sessions, we repeated the linear regression analyses in the subsample with same-day EEG-MRS measurements. As in the main analysis, separate models were run for central and parietal P3b amplitudes, with lDLPFC GABA levels and their interaction with group or cluster as predictors, age and sex as covariates, and bootstrapping applied for robust estimation.

For the group-based regressions, significant lDLPFC GABA × Group interactions were observed at both central (*B* = 10.21, 95% CI [2.19, 17.96], *p* = .010, N_HC_ = 34, N_SSD_ = 18) and parietal sites (*B* = 8.73, 95% CI [1.35, 15.98], *p* = .021, N_HC_ = 34, N_SSD_ = 18). Follow-up within-group regressions showed no significant associations in HC (*p*s > .05), but robust positive associations in SSD (central: *B* = 6.83, 95% CI [3.70, 9.94], *p* < .001; parietal: *B* = 6.57, 95% CI [3.18, 9.89], *p* < .001).

For the cluster-based regressions, significant lDLPFC GABA × Cluster interactions were also found at central (*B* = 9.94, 95% CI [1.09, 18.90], *p* = .029, N_HC_ = 34, N_SSD-_ = 13, N_SSD+_ = 5) and parietal sites (*B* = 8.73, 95% CI [0.68, 17.14], *p* = .032, N_HC_ = 34, N_SSD-_ = 13, N_SSD+_ = 5). Within-group regressions again showed no significant effects in HC (*p*s > .05), but strong positive associations in the SSD- cluster (central: *B* = 6.66, 95% CI [3.31, 10.27], *p* < .001; parietal: *B* = 6.45, 95% CI [2.69, 10.39], *p* = .001).

Together, these results replicate our main findings in the same-day measurement subsample, further supporting the robustness of lDLPFC GABA-centroparietal P3b amplitude associations in SSD, particularly among the lower-symptom cluster (SSD-).

#### Group Differences in Gray Matter Volume of the ACC and lDLPFC

We examined gray matter volume (GMV) differences in the ACC and lDLPFC across groups in our cohort using ANCOVA, with age and sex included as covariates. We included the A46L, A946dL, A946vL, A8vlL, and A9lL regions for the lDLPFC and the A32pL and A32pR regions for the ACC, based on the Human Brainnetome Atlas^11^. No significant differences were observed in lDLPFC GMV (all *p*s > .05). However, ACC volumes were significantly decreased in the SSD group compared to HC (Contrast EMM = −110.30, 95% CI [-207.09, -13.51], *d* = 0.32, *p* = .026), with similar reductions observed in the SSD− (Contrast EMM = −116.60, 95% CI [-226.23, -6.96], *d* = 0.34, *p* = .037); but not in the SSD+ (Contrast EMM = −115.97, 95% CI [-245.12, 13.18], *d* = 0.34, *p* = .078) clusters compared to HC (see [Supplementary Figure 2](#_c6nuksuj1ekv)). No significant differences in ACC volumes were observed between the SSD− and SSD+ clusters (*p* > .05).

# Supplemental Tables

|  | **SSD** | | **HC** | | **p** |
| --- | --- | --- | --- | --- | --- |
|  | **Mean ± SD or n (%)** | **n** | **Mean ± SD or n (%)** | **n** |  |
| **Demographic Characteristics** | | | | | |
| Age, Years | 38.5 ± 13.76 | 58 | 32.55 ± 12.45 | 56 | 0.017^b^ |
| Sex, Female:Male (% Female) | 19:39 (32.8%) |  | 35:21 (62.5%) |  | 0.003^a^ |
| BMI | 26.77 ± 4.9 | 57 | 22.7 ± 3.44 | 55 | < 0.001^b^ |
| Smoking, Yes:No (% Yes) | 26:30 (46.4%) |  | 3:50 (5.7%) |  | < 0.001^a^ |
| **Comorbidities** |  |  |  |  |  |
| Diabetes, Yes:No (% Yes) | 2:56 (3.4%) |  | 0:56 (0%) |  | 0.496^a^ |
| Hypertension, Yes:No (% Yes) | 9:49 (15.5%) |  | 0:56 (0%) |  | 0.003^a^ |
| **Disease Characteristics** | | | | | |
| Disease Duration, Months | 139.71 ± 141.57 | 56 |  |  |  |
| Antipsychotic Treatment Duration,  Months | 107.76 ± 125.8 | 49 |  |  |  |
| Benzodiazepine Use, Yes:No (% Yes) | 12:46 (20.7%) |  |  |  |  |
| CPZeq, mg | 351.27 ± 264.17 | 56 |  |  |  |
| Remission Andreasen, Yes:No (% Yes) | 18:39 (31.6%) |  |  |  |  |
| PANSS Positive Symptoms | 14.11 ± 5.73 | 57 | 7.34 ± 0.96 | 56 | < 0.001^b^ |
| PANSS Negative Symptoms | 14.84 ± 6.11 | 57 | 7.29 ± 0.95 | 56 | < 0.001^b^ |
| PANSS General Symptoms | 31.91 ± 8.82 | 57 | 17.11 ± 1.64 | 56 | < 0.001^b^ |
| PANSS Total Score | 60.86 ± 17.44 | 57 | 31.73 ± 2.92 | 56 | < 0.001^b^ |
| GAF | 51.09 ± 13.46 | 55 | 88.89 ± 7.11 | 56 | < 0.001^b^ |
| BACS Composite z Score | -2.18 ± 1.71 | 53 | 0.15 ± 0.87 | 56 | < 0.001^b^ |
| **Diagnosis** |  |  |  |  |  |
| Schizophrenia | 31 (53.4%) |  |  |  |  |
| Schizoaffective Disorder | 18 (31.0%) |  |  |  |  |
| Brief Psychotic Disorder | 4 (6.9%) |  |  |  |  |
| Unspecified SSD | 4 (6.9%) |  |  |  |  |
| Delusional Disorder | 1 (1.7%) |  |  |  |  |

#### Supplementary Table 1. Sample characteristics table for the ACC MRS measurement. HC = healthy control participant, SSD = schizophrenia spectrum disorder, BACS = Brief Assessment of Cognition in Schizophrenia, BMI = body mass index, CPZeq = chlorpromazine equivalent dose, GAF = Global Assessment of Functioning, PANSS = Positive and Negative Syndrome Scale. *^a^* Fisher’s exact test, *^b^* Welch’s t test.

|  | **SSD** | | **HC** | | **p** |
| --- | --- | --- | --- | --- | --- |
|  | **Mean ± SD or n (%)** | **n** | **Mean ± SD or n (%)** | **n** |  |
| **Demographic Characteristics** | | | | | |
| Age, Years | 38.86 ± 10.31 | 49 | 35.45 ± 11.88 | 51 | 0.129^b^ |
| Sex, Female:Male (% Female) | 13:36 (26.5%) |  | 20:31 (39.2%) |  | 0.206^a^ |
| BMI | 30.43 ± 5.91 | 48 | 24.21 ± 3.08 | 50 | < 0.001^b^ |
| Smoking, Yes:No (% Yes) | 27:22 (55.1%) |  | 5:45 (10%) |  | < 0.001^a^ |
| **Comorbidities** |  |  |  |  |  |
| Diabetes, Yes:No (% Yes) | 2:47 (4.1%) |  | 1:50 (2%) |  | 0.614^a^ |
| Hypertension, Yes:No (% Yes) | 8:41 (16.3%) |  | 4:47 (7.8%) |  | 0.23^a^ |
| **Disease Characteristics** | | | | | |
| Disease Duration, Months | 145.96 ± 96.01 | 49 |  |  |  |
| Antipsychotic Treatment Duration,  Months | 140.52 ± 95.47 | 46 |  |  |  |
| Benzodiazepine Use, Yes:No (% Yes) | 1:48 (2%) |  |  |  |  |
| CPZeq, mg | 333.83 ± 232.96 | 48 |  |  |  |
| Remission Andreasen, Yes:No (% Yes) | 33:16 (67.3%) |  |  |  |  |
| PANSS Positive Symptoms | 10.94 ± 4.15 | 49 | 7.18 ± 0.52 | 51 | < 0.001^b^ |
| PANSS Negative Symptoms | 11.76 ± 4.73 | 49 | 7.57 ± 0.98 | 51 | < 0.001^b^ |
| PANSS General Symptoms | 25.55 ± 7.76 | 49 | 17 ± 1.46 | 51 | < 0.001^b^ |
| PANSS Total Score | 48.24 ± 14.58 | 49 | 31.75 ± 2.31 | 51 | < 0.001^b^ |
| GAF | 56.04 ± 7.78 | 49 | 91 ± 5.59 | 51 | < 0.001^b^ |
| BACS Composite z Score | -1.46 ± 1.44 | 46 | -0.14 ± 1.11 | 48 | < 0.001^b^ |
| **Diagnosis** |  |  |  |  |  |
| Schizophrenia | 36 (73.5%) |  |  |  |  |
| Schizoaffective Disorder | 12 (24.5%) |  |  |  |  |
| Brief Psychotic Disorder | 1 (2.0%) |  |  |  |  |

#### Supplementary Table 2. Sample characteristics table for the lDLPFC MRS measurement. HC = healthy control participant, SSD = schizophrenia spectrum disorder, BACS = Brief Assessment of Cognition in Schizophrenia, BMI = body mass index, CPZeq = chlorpromazine equivalent dose, GAF = Global Assessment of Functioning, PANSS = Positive and Negative Syndrome Scale. *^a^* Fisher’s exact test, *^b^* Welch’s t test.

| **DSM-5 diagnosis** | **N (% of SSD)** | **N (% of SSD-)** | **N (% of SSD+)** |
| --- | --- | --- | --- |
| Schizophrenia | 67 (62.6%) | 39 (60%) | 27 (65.9%) |
| Schizoaffective Disorder | 30 (28.0%) | 19 (29.2%) | 11 (26.8%) |
| Brief Psychotic Disorder | 5 (4.7%) | 3 (4.6%) | 2 (4.9%) |
| Unspecified SSD | 4 (3.7%) | 3 (4.6%) | 1 (2.4%) |
| Delusional Disorder | 1 (0.9%) | 1 (1.5%) | 0 (0%) |

**Supplementary Table 3.** DSM-5 diagnosis subgroup breakdown for SSD (schizophrenia spectrum disorder), SSD− (lower-symptom cluster), and SSD+ (higher-symptom cluster).

|  | **SSD** | | **HC** | | **p** |
| --- | --- | --- | --- | --- | --- |
|  | **Mean ± SD** | **n** | **Mean ± SD** | **n** |  |
| **ACC** | | | | | |
| SNR (diff) | 10.45 ± 3.57 | 29 | 12.24 ± 3.31 | 33 | 0.046^a^ |
| SD% (GABA) | 21.55 ± 4.96 | 29 | 20.15 ± 4.57 | 33 | 0.255 |
| SNR (off) | 14.59 ± 6.79 | 49 | 16.88 ± 5.94 | 51 | 0.076 |
| SD % (Glx) | 15.57 ± 5.2 | 49 | 12.8 ± 4.17 | 51 | 0.004 ^a^ |
| **Left DLPFC** | | | | | |
| SNR (diff) | 26.09 ± 6.59 | 47 | 30.76 ± 7.01 | 51 | 0.001 ^a^ |
| SD% (GABA) | 14.7 ± 3.49 | 47 | 15.37 ± 4.2 | 51 | 0.391 |
| SNR (off) | 36.21 ± 8 | 48 | 40.59 ± 7.73 | 51 | 0.007 ^a^ |
| SD % (Glx) | 9.73 ± 3.89 | 48 | 8.41 ± 2.19 | 51 | 0.043 ^a^ |

**Supplementary Table 4.** MRS quality summary (mean ± SD) and sample sizes (n) for schizophrenia spectrum disorder (SSD) and healthy controls (HC) in anterior cingulate cortex (ACC) and left dorsolateral prefrontal cortex (Left DLPFC). ACC = anterior cingulate cortex, diff = difference spectrum, GABA = gamma-aminobutyric acid, Glx = glutamate + glutamine, lDLPFC = left dorsolateral prefrontal cortex, off = OFF spectrum, SD% = LCModel estimated standard deviations based on Cramer-Rao lower bounds (CRLB), SNR = signal-to-noise ratio. *^a^* Welch’s t test.

| **Group/Cluster Comparison (ANCOVA) Outcomes** | **N_HC_** | **N_SSD_** | **N_SSD−_** | **N_SSD+_** |
| --- | --- | --- | --- | --- |
| ACC GABA (MRS) | 33 | 29 | 14 | 14 |
| lDLPFC GABA (MRS) | 51 | 47 | 37 | 10 |
| ACC Glx (MRS) | 51 | 49 | 23 | 25 |
| lDLPFC Glx (MRS) | 51 | 48 | 38 | 10 |
| Frontal P3b amplitude & latency | 101 | 102 | 62 | 39 |
| Central P3b amplitude & latency | 104 | 106 | 65 | 40 |
| Parietal P3b amplitude & latency | 106 | 107 | 65 | 41 |
| **Metabolite–**P3b **Regressions** | **N_HC_** | **N_SSD_** | **N_SSD−_** | **N_SSD+_** |
| Frontal P3b Amplitude ~ ACC GABA × Group/Cluster | 32 |  | 13 | 14 |
| Frontal P3b Amplitude ~ lDLPFC GABA × Group/Cluster | 49 |  | 35 | 8 |
| Central P3b Amplitude ~ ACC GABA × Group/Cluster | 32 | 29 | 14 | 14 |
| Central P3b Amplitude ~ lDLPFC GABA × Group/Cluster | 51 | 46 | 37 | 9 |
| Parietal P3b Amplitude ~ ACC GABA × Group/Cluster | 32 | 29 | 14 | 14 |
| Parietal P3b Amplitude ~ lDLPFC GABA × Group/Cluster | 51 | 47 | 37 | 10 |
| Frontal P3b Amplitude ~ ACC Glx × Group/Cluster | 48 |  | 22 | 25 |
| Frontal P3b Amplitude ~ lDLPFC Glx × Group/Cluster | 49 |  | 36 | 8 |
| Central P3b Amplitude ~ ACC Glx × Group/Cluster | 48 | 49 | 23 | 25 |
| Central P3b Amplitude ~ lDLPFC Glx × Group/Cluster | 51 | 47 | 38 | 9 |
| Parietal P3b Amplitude ~ ACC Glx × Group/Cluster | 50 | 49 | 23 | 25 |
| Parietal P3b Amplitude ~ lDLPFC Glx × Group/Cluster | 51 | 48 | 38 | 10 |
| **P3b & MRS ~ Cognition &Behavior Correlations (SSD/SSD− only)** | **N_SSD_** | **N_SSD−_** |  |  |
| Central P3b Amplitude ~ BACS composite z-score | 98 | 61 |  |  |
| Central P3b Amplitude ~ Reaction time | 106 | 65 |  |  |
| Central P3b Amplitude ~ Task accuracy | 106 | 65 |  |  |
| Central P3b Amplitude ~ CPZeq | 103 | 62 |  |  |
| Parietal P3b Amplitude ~ BACS composite z-score | 99 | 61 |  |  |
| Parietal P3b Amplitude ~ Reaction time | 107 | 65 |  |  |
| Parietal P3b Amplitude ~ Task accuracy | 107 | 65 |  |  |
| Parietal P3b Amplitude ~ CPZeq | 104 | 62 |  |  |
| lDLPFC GABA ~ BACS composite z-score | 44 | 34 |  |  |
| lDLPFC GABA ~ Reaction time | 47 | 37 |  |  |
| lDLPFC GABA ~ Task accuracy | 47 | 37 |  |  |
| lDLPFC GABA ~ CPZeq | 46 | 36 |  |  |

**Supplementary Table 5.** Sample breakdown for all group/cluster comparisons, metabolite–P3b regressions, and P3b/MRS–cognition/behavior correlation analyses. All analyses included Age and Sex as covariates. Correlation analyses were performed in SSD or SSD− groups only, as indicated. Empty cells indicate groups that were not carried forward in the stepwise pipeline (e.g., no group-based regression was conducted when no differences were found at the ANCOVA stage). ACC = anterior cingulate cortex, BACS = Brief Assessment of Cognition in Schizophrenia, CPZeq = chlorpromazine equivalent dosage, GABA = gamma-aminobutyric acid, Glx = glutamate + glutamine, HC = healthy controls, lDLPFC = left dorsolateral prefrontal cortex, MRS = magnetic resonance spectroscopy, SSD = schizophrenia spectrum disorder.

# Supplemental Figures


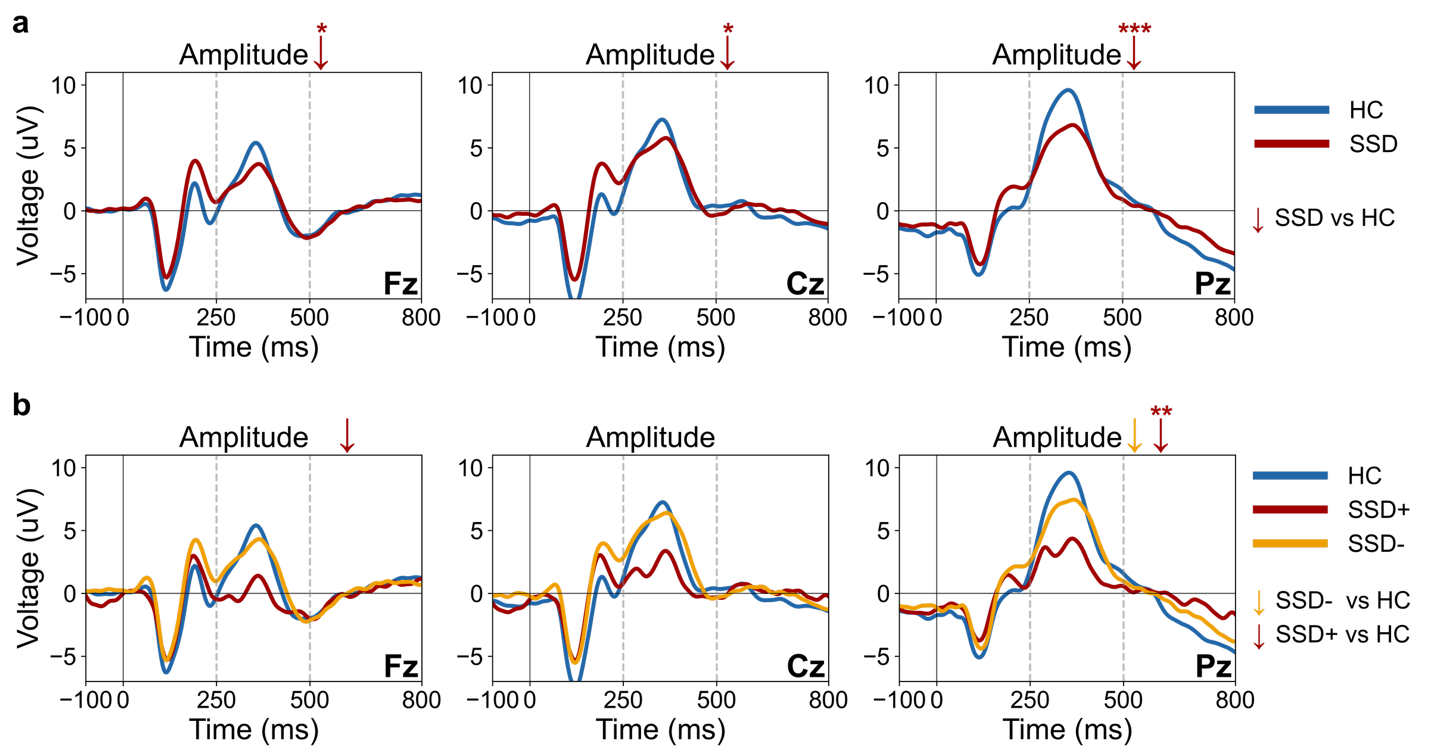


#### Supplementary Figure 1. Fz, Cz, and Pz ERPs showing P3b amplitude and latency differences across: (a) HC and SSD groups, and (b) HC, SSD-, and SSD+ clusters. The red (SSD, SSD+) and yellow (SSD-) arrows (↓) show the decrease in amplitude compared to HC, with arrows without asterisks indicating trend-level effects (*p_FDR_* < .1). HC = healthy controls, SSD = schizophrenia spectrum disorder, SSD- = lower-symptom cluster, SSD+ = higher-symptom cluster, ms = milliseconds, μV = microvolts. Significance level: * < .05, ** < .01, *** < .001.

####
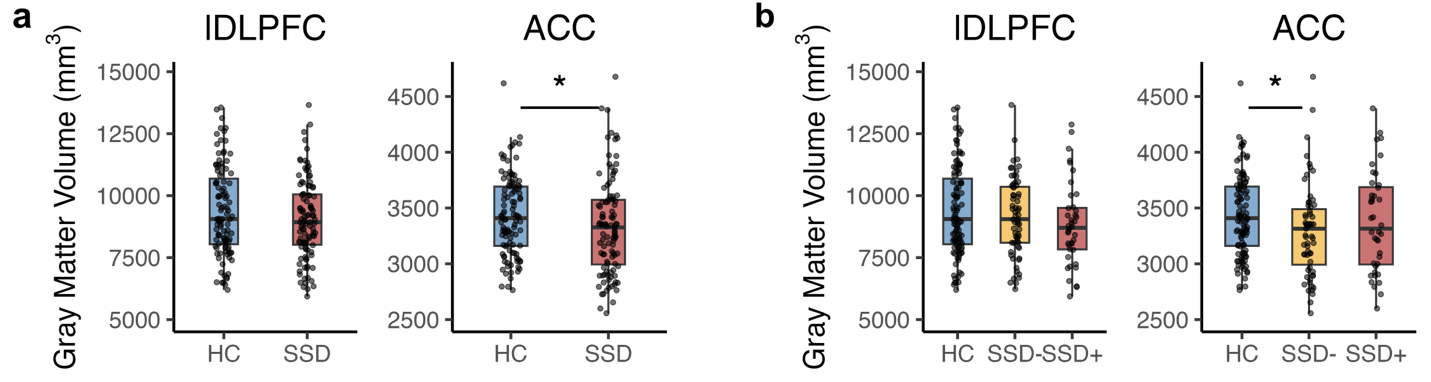


#### Supplementary Figure 2. ACC and lDLPFC gray matter volumes across: (a) HC and SSD groups, and (b) HC, SSD-, and SSD+ clusters. HC = healthy controls, SSD = schizophrenia spectrum disorder, SSD- = lower-symptom cluster, SSD+ = higher-symptom cluster, ACC = anterior cingulate cortex, lDLPFC = left dorsolateral prefrontal cortex, mm^3^ = cubic millimeters. Box-plot elements are defined as follows: the center line represents the median, the box limits indicate the upper and lower quartiles (25th and 75th percentiles), and the whiskers extend to 1.5 times the interquartile range (IQR). Each point represents individual subject data. Significance level: * < .05, ** < .01, *** < .001.


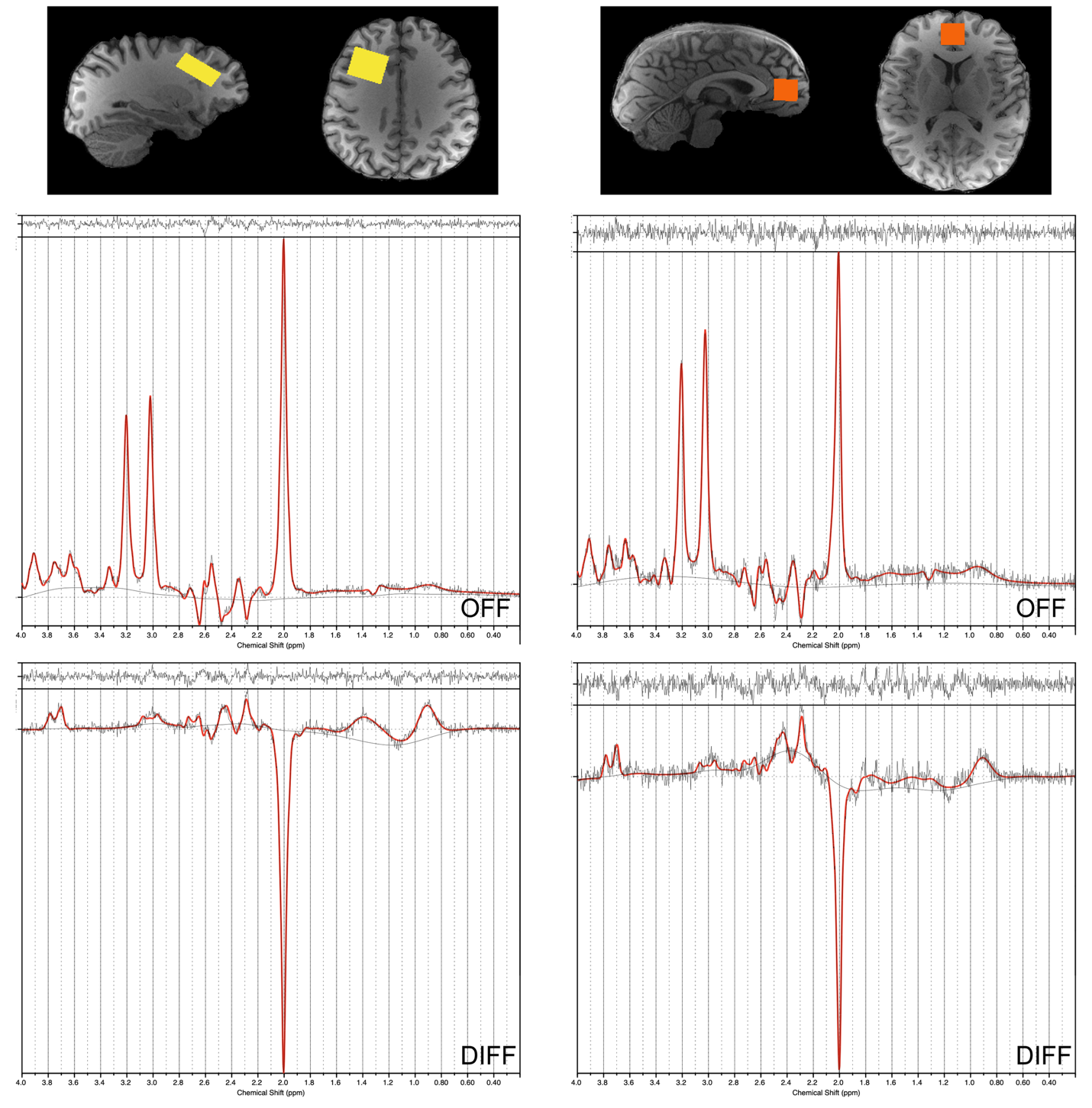


**Supplementary Figure 3.** Representative voxel placement and corresponding spectra for the left dorsolateral prefrontal cortex (lDLPFC) on the left side and anterior cingulate cortex (ACC) on the right side. Shown are anatomical locations of the MRS voxels overlaid on structural T1-weighted images, along with examples of OFF (unedited; for Glx) and DIFF (edited; for GABA) spectra and LCModel fits.

# Supplemental References

1. Kay SR, Fiszbein A, Opler LA. The Positive and Negative Syndrome Scale (PANSS) for Schizophrenia. *Schizophr Bull*. 1987;13(2):261-276. doi:10.1093/schbul/13.2.261

2. Andreasen NC, Carpenter WT, Kane JM, Lasser RA, Marder SR, Weinberger DR. Remission in Schizophrenia: Proposed Criteria and Rationale for Consensus. *Am J Psychiatry*. 2005;162(3):441-449. doi:10.1176/appi.ajp.162.3.441

3. Leucht S, Samara M, Heres S, Davis JM. Dose Equivalents for Antipsychotic Drugs: The DDD Method: Table 1. *Schizophr Bull*. 2016;42(suppl 1):S90-S94. doi:10.1093/schbul/sbv167

4. Heatherton TF, Kozlowski LT, Frecker RC, Fagerstrom K. The Fagerström Test for Nicotine Dependence: a revision of the Fagerstrom Tolerance Questionnaire. *Br J Addict*. 1991;86(9):1119-1127. doi:10.1111/j.1360-0443.1991.tb01879.x

5. Keefe RSE, Goldberg TE, Harvey PD, Gold JM, Poe MP, Coughenour. The Brief Assessment of Cognition in Schizophrenia: reliability, sensitivity, and comparison with a standard neurocognitive battery. *Schizophr Res*. 2004;68(2-3):283-297. doi:10.1016/j.schres.2003.09.011

6. Sachs G, Winklbaur B, Jagsch R, Keefe RSE. Validation of the German Version of the Brief Assessment of Cognition in Schizophrenia (BACS) – Preliminary Results. *Eur Psychiatry*. 2011;26(2):74-77. doi:10.1016/j.eurpsy.2009.10.006

7. Winkler I, Haufe S, Tangermann M. Automatic Classification of Artifactual ICA-Components for Artifact Removal in EEG Signals. *Behav Brain Funct*. 2011;7(1):30. doi:10.1186/1744-9081-7-30

8. Ford JM, Roach BJ, Hoffman RS, Mathalon DH. The dependence of P300 amplitude on gamma synchrony breaks down in schizophrenia. *Brain Res*. 2008;1235:133-142. doi:10.1016/j.brainres.2008.06.048

9. Ergen M, Marbach S, Brand A, Başar-Eroğlu C, Demiralp T. P3 and delta band responses in visual oddball paradigm in schizophrenia. *Neurosci Lett*. 2008;440(3):304-308. doi:10.1016/j.neulet.2008.05.054

10. Gallinat J, Kunz D, Senkowski D, et al. Hippocampal glutamate concentration predicts cerebral theta oscillations during cognitive processing. *Psychopharmacology (Berl)*. 2006;187(1):103-111. doi:10.1007/s00213-006-0397-0

11. Fan L, Li H, Zhuo J, et al. The Human Brainnetome Atlas: A New Brain Atlas Based on Connectional Architecture. *Cereb Cortex*. 2016;26(8):3508-3526. doi:10.1093/cercor/bhw157
